# First step results from a phase II study of a dendritic cell vaccine in glioblastoma patients (CombiG-vax)

**DOI:** 10.3389/fimmu.2024.1404861

**Published:** 2024-08-13

**Authors:** Laura Ridolfi, Lorena Gurrieri, Nada Riva, Jenny Bulgarelli, Francesco De Rosa, Massimo Guidoboni, Valentina Fausti, Nicoletta Ranallo, Sebastiano Calpona, Marcella Tazzari, Massimiliano Petrini, Anna Maria Granato, Elena Pancisi, Flavia Foca, Monia Dall’Agata, Isabella Bondi, Elena Amadori, Pietro Cortesi, Chiara Zani, Valentina Ancarani, Alessandro Gamboni, Antonio Polselli, Giuseppe Pasini, Daniela Bartolini, Giuseppe Maimone, Donatella Arpa, Luigino Tosatto

**Affiliations:** ^1^ Experimental and Clinical Oncology of Immunotherapy and Rare Cancers, Biobank Unit, IRCCS Istituto Romagnolo per lo Studio dei Tumori (IRST) “Dino Amadori”, Meldola, Italy; ^2^ Unit of Biostatistics and Clinical Trials, IRCCS Istituto Romagnolo per lo Studio dei Tumori (IRST) “Dino Amadori” Meldola, Meldola, Italy; ^3^ Radiology Unit, IRCCS Istituto Romagnolo per lo Studio dei Tumori (IRST) “Dino Amadori” Meldola, Meldola, Italy; ^4^ Cardioncology Division, IRCCS Istituto Romagnolo per lo Studio dei Tumori (IRST) “Dino Amadori”, Meldola, Italy; ^5^ Pharmacy Unit, IRCCS Istituto Romagnolo per lo Studio dei Tumori (IRST) “Dino Amadori”, Meldola, Italy; ^6^ Department of Oncology and Hematology AUSL Romagna, Lugo, Italy; ^7^ Oncology Department Cattolica Hospital, Cattolica, Italy; ^8^ Oncology Department Rimini Hospital, Rimini, Italy; ^9^ Pathology Unit, “Maurizio Bufalini” Hospital, Cesena, Italy; ^10^ Neurosurgery Unit Maurizio Bufalini Hospital, Cesena, Italy; ^11^ Radiotherapy Unit, IRCCS Istituto Romagnolo per lo Studio dei Tumori (IRST) "Dino Amadori", Meldola, Italy

**Keywords:** glioblastoma, vaccine, immunotherapy, dendritic cell, adoptive cell therapy, radiochemotherapy

## Abstract

**Background:**

Glioblastoma (GBM) is a poor prognosis grade 4 glioma. After surgical resection, the standard therapy consists of concurrent radiotherapy (RT) and temozolomide (TMZ) followed by TMZ alone. Our previous data on melanoma patients showed that Dendritic Cell vaccination (DCvax) could increase the amount of intratumoral-activated cytotoxic T lymphocytes

**Methods:**

This is a single-arm, monocentric, phase II trial in two steps according to Simon’s design. The trial aims to evaluate progression-free survival (PFS) at three months and the safety of a DCvax integrated with standard therapy in resected GBM patients. DCvax administration begins after completion of RT-CTwith weekly administrations for 4 weeks, then is alternated monthly with TMZ cycles. The primary endpoints are PFS at three months and safety. One of the secondary objectives is to evaluate the immune response both *in vitro* and *in vivo* (DTH skin test).

**Results:**

By December 2022, the first pre-planned step of the study was concluded with the enrollment, treatment and follow up of 9 evaluable patients. Two patients had progressed within three months after leukapheresis, but none had experienced DCvax-related G3-4 toxicities Five patients experienced a positive DTH test towards KLH and one of these also towards autologous tumor homogenate. The median PFS from leukapheresis was 11.3 months and 12.2 months from surgery.

**Conclusions:**

This combination therapy is well-tolerated, and the two endpoints required for the first step have been achieved. Therefore, the study will proceed to enroll the remaining 19 patients. (Eudract number: 2020-003755-15 https://www.clinicaltrialsregister.eu/ctr-search/trial/2020-003755-15/IT)

## Introduction

Glioblastoma (GBM) is a poor prognosis malignant WHO grade 4 glioma whose standard therapy, after surgical resection, consists of radio-therapy (RT) and chemotherapy (CT) with temozolomide (TMZ) according to Stupp et al. However, the prognosis remains poor with a 5% five-year survival (1). In recent years, there has been renewed interest in immunotherapy of cancer due to new drugs and effective therapies, such as immune checkpoints inhibitors (ICIs), adoptive T-cell approaches, dendritic cell-based vaccines, or combinations of these. Although studies with active immunotherapy have used different types of molecules, their results have not been consistent enough to be approved by the FDA (2). GBM has an immunosuppressive microenvironment due to tumor-associated factors: overexpression of inhibitory cytokines or checkpoint molecules, low levels of HLA expression on tumor cells, and an abundance of infiltrating regulatory T cells (Treg) (3). Thus, it remains an aggressive cancer with limited therapeutic options due to the interactions between tumor cells and the microenvironment, which require more targeted agents against both components (4). Dendritic cells (DCs) are the most potent professional antigen-presenting cells due to their linking function between innate and adaptive immune responses, becoming a promising way to generate a specific immune response against cancer (5). Regarding the use of dendritic cell vaccination in HGGs (High-grade gliomas), numerous studies have been published and are underway to evaluate the safety and efficacy of DC-based vaccines in GBM patients (6, 7). Moreover, in 2014 two meta-analyses were published, indicating improved survival (OS) and progression-free survival (PFS) with a DC vaccination in HGG patients. In 2023 Oster et al. identified the main phase III clinical trials for adult GBM and among these, he cited Kong because his study investigated cytokine-induced killer (CIK) cells combined with standard radio-chemotherapy which prolonged PFS (8.1 months) (8, 9). Moreover, a two most recent meta-analysis were relevant to demonstrate a different outcome in GBM patients treated with DC vaccination in terms of OS and PFS. The authors agreed on the safety, in fact they didn’t report severe adverse events (AEs), regardless of the number of cycles, dosages and the administration route (10, 11). Finally, a phase III trial by Liau et al. found that OS was longer in the arm treated with their autologous DC vaccine compared to the standard therapy (12).They randomized the 331 patients to receive DCVax-L plus temozolomide vs placebo plus temozolomide; in case of disease progression/relapse during treatment, crossover was allowed. Considering this, our study is monocentric and all patients after surgery had received the same treatment because the placebo was not expected.

### Study rationale

Since 2001, we have treated more than 80 advanced melanoma patients with a tumor homogenate-loaded autologous DC vaccine, and we observed a clinical benefit of 54.1%. The results showed that, in patients who develop an immune response to the vaccination antigens (about two-thirds), OS is significantly improved compared to other patients and advanced melanoma patients treated with chemotherapy (13). In a past trial we evaluated the DCvax combined with low-dose TMZ aimed at reducing the number of Tregs (14), and now more clinical trials are ongoing. All the studies on melanoma patients have confirmed that the DCvax has a good safety profile, similar to published studies by others (2, 6, 7).

Our results show that the DCvax has a crucial role in promoting intra-tumoral T cell activation seen in post-therapy lesions, which is mitigated by concurrent up-regulation of PDL1 and the occurrence of adaptive immunological resistance (15).

Based on our previous data, we outlined the main reasons for the design of this study:

1. After resection and RT-CT patients are in a state of minimal residual disease, which is beneficial for immunotherapy because of the lower tumor load and depletion of immunosuppressive cells
2. Our DCvax is safe
3. In patients who develop an immune response after DCvax OS is significantly improved
4. DCvax increases intra-tumoral T cell infiltration and activation

- TMZ reduces T regs
- The lymphocyte compartment recovery post-chemotherapy appears to be beneficial for the induction of anti-tumor responses
- Dying tumor cells after RT-CT may act as a warning signal and boost an effective antitumor immune response
- An increased responsiveness to TMZ is seen after DCvax

## Materials and methods

The study is divided into two parts: a pre-screening phase and a main study. Pre-screening and patient enrollment occur after surgery. As surgery is a clinical activity, it is not part of the study. During the pre-screening phase, patients undergo procedures necessary to obtain the biological material needed for DCvax manufacturing (leukapheresis) and are treated with standard RT-CT (according to the Stupp regimen).

After the pre-screening phase, patients are enrolled based on the following

Inclusion Criteria:

1. Histologically confirmed glioblastoma.
2. The autologous surgical specimen needed for vaccine manufacturing must have been collected and sent to the Somatic Cell Therapy Lab of IRCCS IRST and must fulfill the criteria prescribed by the GMP procedures.
3. Availability of sufficient leukapheresis material for the preparation of the DCvax.
4. Patients must have recovered (grade 1 or less by CTCAE 5.0) from all the events related to previous treatments.
5. Provide written informed consent/assent for the trial.
6. Be ≥ 18 years of age on the day of signing informed consent.
7. Have a Karnofsky performance status (KPS) ≥ 70% or a performance status of 0 or 1 on the ECOG Performance Scale.
8. Demonstrate adequate organ and marrow function.

Then the main exclusion criteria are:

1. Patients diagnosed with immunodeficiency or receiving systemic steroid therapy > 20 mg prednisolone equivalent (or 3 mg dexamethasone equivalent), or any other form of immunosuppressive therapy within seven days before the first dose of trial treatment.
2. Patients with an active autoimmune disease that has required systemic treatment in the past two years (i.e. using disease-modifying agents, corticosteroids, or immunosuppressive drugs). Replacement therapy (e.g., thyroxine, insulin, or physiologic corticosteroid replacement therapy for adrenal or pituitary insufficiency, etc.) is not considered a form of systemic treatment.
3. Known history of active TB (Bacillus Tuberculosis).
4. Previous treatment with a cancer vaccine.
5. Other known malignant neoplastic diseases in the patient’s medical history with a disease-free interval of less than five years, except basal or squamous cell carcinoma of the skin and *in situ* carcinoma of the cervix uteri treated with radical surgery.
6. Any known history of, or current serologic marker positivity indicating infection by Treponema pallidum, hepatitis B virus (HBsAg, HbsAb, HbcAb), hepatitis C virus (HCVAb, HCV RNA quantitative), human immunodeficiency virus (HIV), whether actual or previous.
7. Patients who had received a live vaccine within 30 days of the study’s scheduled starting date.

**Figure 1:**
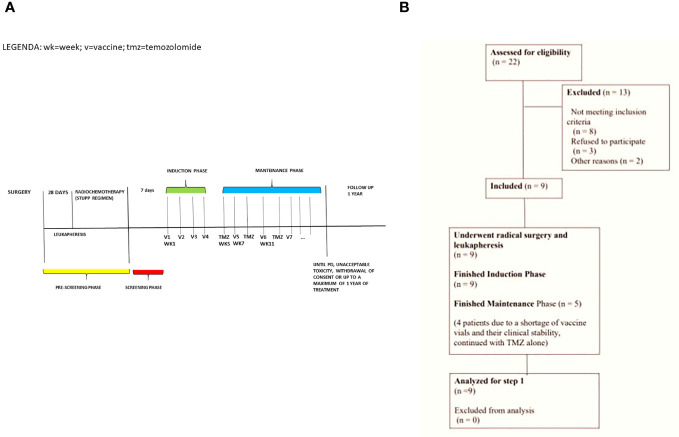
**(A)** Study Schema: pre-screening phase of 28 days, screening phase of 7 days during which the baseline DTH skin test is performed, induction phase of 4 weeks, and maintenance phase lasting up to 11 months. **(B)** Consort Flow Diagram summarizing screening failures.

The experimental treatment consists of an induction phase with four-weekly doses of the DCvax (1.0 × 10^7^ cells), administered intradermally (weeks one to four), followed by a maintenance phase consisting of 28-day cycles with vaccine administration at the start of week seven, and TMZ (150-200mg/m2/day) assumed orally from day one to five q28 (starting week five). The combined maintenance treatment continues until disease progression, unacceptable toxicity, the patient withdrawing consent, or until the maximum of one-year treatment time (**Figure 1A**). To evaluate response and progression, we used the international Response Evaluation Criteria from the Brain Tumors Committee (Response Assessment in Neuro-Oncology guidelines, RANO), associated with Perfusion MRI by DSC (dynamic susceptibility contrast). Our objective was to distinguish between the real progression of GBM from radionecrosis and pseudo-progression by evaluating the five ROIs (Region Of Interest) of the perfusion study and correlating them with RANO imaging criteria. In particular, we wanted to demonstrate that the presence in at least three ROIs of rCBV (Relative Cerebral Blood Volume) values> 2.5 identifies real disease progression according to the RANO imaging criteria.

For this study, patients are reevaluated for response at the end of the induction phase and every two cycles during the maintenance phase (16). (Protocol in **Supplementary Documents**)

### DC vaccine preparation

The “autologous dendritic cell loaded with autologous tumor homogenate” is an Advanced Therapy Medicinal Product consisting of dendritic cells obtained by *in vitro* differentiation of the peripheral blood monocytes. DCs are generated *in-vitro* from circulating precursors obtained by leukapheresis without previous mobilization. Peripheral blood mononucleated cells (PBMCs) are purified by Ficoll gradient centrifugation and cultured in flasks for 2 h. Then, non adherent cells are discarded, whereas adherent cells are cultured in a medium supplemented with recombinant human interleukin (IL)-4 and GMCSF for 6 days to allow them to differentiate into immature DCs. On day 6 immature DCs are pulsed for 16-20 hours to induce uploading of tumor antigens with autologous tumor homogenate at the dose of 100 μg/ml of culture (90-95% of culture volume) and keyhole limpet haemocyanin (KLH) at the dose of 50 μg/ml of culture (5-10% of culture volume) and matured with a cytokine cocktail containing IL1b, PGE2, IL6, and TNF-a (“maturation cocktail”). Pulsed mature dendritic cells (mDC) are collected on day nine, washed, counted, and tested for quality control (vitality, purity, phenotype markers, sterility, endotoxin, mycoplasma), frozen in aliquots (at least 13×10^6^ cells/aliquot), and stored in nitrogen vapors. The aliquots are thawed and inserted into two insulin syringes for administration to the patient (10x10^6^ total dendritic cells). The syringes are filled and closed in the Class A area. Subsequently, the syringes are packed, labeled, and put in a plastic bag reporting the product and the protocol identity.

The patient receives five intradermal injections in locations near the lymph nodes that have not undergone surgery before (for any reasons), alternating injection sites for each vaccine administration.

### DTH test

*In vivo* immunomonitoring is measured by the delayed-type hypersensitivity (DTH) test against tumor homogenate and KLH. DTH test is a classical method for measuring cell-mediated immune reactivity and involves intradermal administration of antigen preparation followed by the recording of the degree of erythema and induration produced 24-48 hours after the injection. The response reflects antigen-specific recruitment and activation of CD4+ to release T helper-1 cytokines (IFN-γ in particular) and the recruitment and activation of CD8+ effector T cells in the injection site. In our and other groups’ experience, a positive response to the DTH test performed with soluble antigens after DCvax in patients carrying metastatic melanoma was strongly related to better clinical outcomes. In addition, DTH testing neither requires extensive training nor costly equipment, and it can be performed at the bedside, making it a feasible, low-cost immunomonitoring method to evaluate immunologic efficacy in a clinical trial setting. The DTH testing dose corresponds to 50 μg of autologous tumor homogenate or KLH (positive control) prepared in 0.5 ml of 0.9% sterile saline. The negative control is a dose of 0.5 ml of 0.9% sterile saline alone. The diameter of induration/erythema observed after 24 hrs is recorded according to the following scale: 0-5 mm grade 1 (+), 6-10 mm grade 2 (++), 11-20 mm grade 3 (+++), > 21 mm grade 4 (++++).

### Objectives and statistical considerations

The main objectives of the study are to measure Progression-Free Survival (PFS), which aims to identify the proportion of patients who do not experience disease progression three months after their leukapheresis date, and to ensure patient safety by recording the percentage of patients who experience grade 3 or higher adverse events related to the treatment.

The secondary objectives are to evaluate the immune response *in vivo* by analyzing the prognostic role of a positive DTH skin test after treatment and, additionally, to measure the clinical outcome in terms of OS and the efficacy of the treatment to enhance the number of circulating immune effectors specific for tumor antigens. To this aim the persistence of anti-tumor immune response will be also evaluated as well as the plasma levels of proangiogenic factors and of inflammatory cytokines. Moreover, the prognostic and predictive role of the tumor antigen expression and of infiltrated immune cells will also be analyzed in tumor tissue.

Simon’s two-stage design (17) was used for the sample size calculation. The null hypothesis that 70% of patients did not experience disease progression three months after leukapheresis was tested against a one-sided alternative.

1. In order to complete stage 1 of the study, we enrolled nine patients as pre-planned. Three months after the start of leukapheresis, at least six patients showed no signs of disease progression.
2. To complete stage 2 (and the entire trial), we will enroll 19 additional patients for a total of 28 (stage 1 + stage 2). If more than 22 out of 28 patients show no evidence of disease progression at three months, the null hypothesis will be rejected. This design yields a 10% false positive rate and an 80% true positive rate for an 87% true proportion.

The calculation determines the percentage of patients who did not experience progression within three months, which is also the percentage of patients achieving disease control rate at the same time point. Furthermore, non-progressive patients are monitored from the date of leukapheresis to the date of first progression, the date of death from any cause, or the date of the last restaging. The OS is measured from the date of leukapheresis until the date of death from any cause or the last known date the patient was alive.

PFS and OS are calculated with the Kaplan-Meier method, and the analysis is carried out on the eligible population. The percentage of patients experiencing vaccine-related G≥ 3 adverse events (AEs) during the treatment will be inferred using the two-sided Clopper-Pearson, or a more appropriate one, with a 95% confidence interval. Descriptive statistics are used to assess the extent of the secondary endpoints.

The pre-planned interim analysis for the safety and efficacy of the first step has been concluded.

## Results

In December 2022, nine patients were evaluated for safety and efficacy, allowing the pre-planned analysis to be performed. Screening failure occurred in 13 patients: for 8 patients, we did not collect enough tumor tissue to ensure that we could perform at least 75% of the induction treatment (3 out of 4 planned vaccines), 3 patients withdrew consent, 2 patients had a rapid worsening of their clinical condition (**Figure 1B**).

All the patients (5 males and 4 females) with a median age of 58 years (range: 58-69) underwent radical surgery and leukapheresis before starting RT-CT. Four out of 9 patients, due to a shortage of vaccine vials and their clinical stability, continued with TMZ alone for the maintenance phase. However, all patients completed the induction phase, a necessary condition for them to be considered evaluable by protocol and after which the first re-evaluation was planned.

One week after completing RT-CT, they began the vaccine induction phase. **Table 1** describes patients characteristics. The median vaccine cycle was six (range: 3-12).At baseline DTH skin test was negative in all nine patients, but after the induction phase it turned out positive on KLH test alone in four patients and on both KLH and tumor Homogenate in one patient. In contrast, we did not observe any positivity on tumor homogenate test alone. Finally the DTH test result correlated neither with MGMT status nor with residual disease post surgery.

**Table 1:** Patient’s characteristics.

Pat.ID	Resection status	MGMT* met %	Site of primary	N° of vaccine cycle (I/M)	DTH test H/KLH §
#0001	subtotal	53%	F-P lobe	4/1	0/++
#0002	subtotal	22%	T-O lobe	4/5	+/+++
#0003	subtotal	21%	F-T lobe	4/2 vial shortage	0/0
#0004	gross	0	P lobe	4/2 vial shortage	0/0
#0005	subtotal	13%	F Lobe	4/1	0/+
#0006	gross	0%	T lobe	4/5	0/++
#0007	gross	49%	F lobe	3/0 vial shortage	0/0
#0008	gross	0%	F lobe	4/8	0/0
#0009	subtotal	0%	T lobe	4/5 vial shortage	0/+++

MGMT met, MGMT methylation; F, frontal; P, parietal; T, temporal; I/M induction/Maintenance; DTH, delayed-type hypersensitivity; H, Homogenate; KLH, Keyhole limpet hemocyanin; * tested by Pyrosequencing § at baseline DTH test was negative for both H and KLH in all nine patients.

The diameter of induration/erythema observed after 24 hrs is recorded according to the following scale: 0-5 mm grade 1 (+), 6-10 mm grade 2 (++), 11-20 mm grade 3 (+++), > 21 mm grade 4 (++++).

All 9 patients were evaluable for AE analysis (patients who had at least 30 days of observation after first vaccine administration). **Table 2** summarizes the targeted AEs reported by AE type and maximum grade. All G3-4 toxicities observed were related to TMZ and in line with the drug’s expected ones. As shown in **Table 3** two patients progressed within three months after leukapheresis. At the cut-off date, all patients were alive except one and were being treated with TMZ. However, the patients who had shown disease progression according to study criteria but had clinical benefit and had not a clinical significant progression in MRI continued the TMZ treatment outside the study. None of these patients had yet to start a second line treatment. At the start of the induction phase, the median amount of dexamethasone consumed by patients, during all the entire treatment, was 2 milligrams (prednisolone equivalent dose 13 mg), which gradually reduced during the four weeks of treatment (compatible with the patient’s symptoms). Seven out of nine patients had a functional perfusion evaluation done because of a pathologic enhancement, whereas two patients had insufficient residual disease for at least three consecutive controls, making the functional perfusion study unnecessary. Of the latter, one patient underwent 10 DCvax cycles and the other underwent 12 DCvax cycles, achieving long-term disease control. At the end of the study, a correlation will be performed between the functional study, immunoRANOs (18), RANOs, and clinical outcomes to meet the exploratory objectives. As of June 2023, the median follow-up after surgery was 13.8 months (range: 13.3-24.7 months). The median PFS from leukapheresis was 11.3 months (95%CI: 3.2-Not estimable) and 12.2 months from surgery (95%CI: 4.7-Not estimable). The median OS from leukapheresis was 23.1 months and 24.7 from surgery (95%:13.0-Not estimable) (**Table 3**).

**Table 2:** Treatment-related adverse events.

AE	N° of patients (%)*			
	G1	G2	G3	G4
**Asthenia**	1 (11,1)	0	0	0
**Fatigue**	1 (11,1)	0	0	0
**Local reaction at vaccine**	3 (33,3)	0	0	0
**Nausea#**	1 (11,1)	1 (11,1)	0	0
**Neutropenia#**	1 (11,1)	0	0	1 (11,1)
**Pain, specify**	1 (11,1)	0	0	0
**Pruritus, spec if gen**	3 (33,3)	1 (11,1)	0	0
**Redness in site of injection**	1 (11,1)	0	0	0
**Skin, specify**	1 (11,1)	0	0	0
**Thrombocytopenia#**	0	0	1 (11,1)	0
**Constipation**	1 (11,1)	0	0	0
**Hypokalemia**	0	1 (11,1)	0	0

#related to temozolomide

*Number of toxicity/percentage of toxicity calculated on 9 patients

**Table 3:** Median follow up and patient’s clinical outcomes.

		N° patients	Median value
Median follow up			(min-max)
*From surgery*		*9*	*13.8 (13.3-24.7)*
*From leukapheresis*		*9*	*12.9 (12.2-23.2)*
**Clinical Outcomes**			*Median value (95%CI)*
*Progression-free survival*			
	*From surgery*	*9*	*12.2 (4.7-NE)*
	*From leukapheresis*	*9*	*11.3 (3.2-NE)*
*Overall survival*			
	*From surgery*	*9*	*24.7 (13.0-NE)*
	*From leukapheresis*	*9*	*23.1 (11.9-NE)*

NE, not estimable from statistical software due to small number of cases.

## Discussion

GBM has a poor prognosis with a median survival of approximately 12-14 months and less than a 5% five-year survival, even when patients receive the Stupp regimen treatment post-surgery. Nowadays, the only prognostic factor related to the outcome remains the methylation status of the O6-methylguanine-DNA methyltransferase (MGMT) gene promoter. The cut off value of methylation cited in literature is higher than 9% and may be a predictive marker of sensitivity to alkylating agents (19). In January 2023, Liau et al. published a phase III trial. They compared OS between the Stupp regimen (concomitant RT and TMZ followed by TMZ), and the same treatment associated with DC vaccine. Three hundred thirty-one patients were enrolled. The trial raised a lot of criticism regarding its methodology, not only because it removed PFS from the primary endpoint but because of its *post hoc* introduction of “control patients” by pooled external control data (20). Despite these limitations, the trial outcome showed evidence of a statistically significant longer OS for patients receiving the combination therapy. In addition to their primary endpoint of OS, they conducted an exploratory analysis on biomarkers and immunogenicity that may correlate with OS and responses to the DC vaccine. Similar results were reported in the Lepski et al. study, which investigated the difference between a combination treatment and an allogeneic DC vaccine after a standard RT-CT regimen. In recurrent patients, the vaccination leads to an OS of 27.6 months (21). These recent trials and our first-step results emphasize the potential impact of immunotherapy, particularly DC cell-based approaches concerning patients with a poor prognosis. Our previous data (on melanoma patients) demonstrated that TMZ could selectively reduce the circulating T regs, and DCvax may favor objective responses to subsequent therapies (14, 22). In melanoma patients we also demonstrated that patients who developed *in vivo* immune responses (DTH skin test positivity after treatment) gained a survival advantage (13). Based on the data, we suggest combining the vaccine with standard therapies to increase drugs’ synergistic effect. To understand the full impact of the vaccine, we added some exploratory objectives, including a functional radiological study of perfusion to correlate with iRANOs, immunological responses, and clinical outcomes. A complete analysis of all objectives will be performed upon completion of the study. Out of the first nine patients, we observed that two patients receiving treatment for one year, with 10 and 12 DCvax cycles, respectively, had minimal residual disease, which didn’t require any further ROI evaluation. Furthermore, they had three consecutive negative radiological controls, making the functional perfusion study unnecessary. The study has very restrictive radiological criteria to determine progression, resulting in the exit of patients from the study. To prioritize patient safety and maintain conservative approach, continuation of treatment beyond progression is not planned by study. However, 8 out of 9 patients, due to clinical benefit and to marginal radiological disease progression confirmed during multidisciplinary discussion, continued TMZ treatment outside the study. Only 1 patient out of 9 proceeded immediately to second-line therapy. The DCvax treatment was able to induce *in vivo* immune response in 5 patients, which we saw was associated with improved outcome in melanoma patients. The main limitation of our study lies in the vaccine manufacture that has to comply with Good Manufacturing Practice (GMP) rules. Furthermore, our vaccine is entirely autologous, and the main reason for the screening failure was the limited quantity of tumor tissue that was available, which prevented us from producing enough vaccine vials. On the other hand, the excellent toxicity profile, the capacity to elicit immunological responses, and the easy route of administration are strengths. The advantage of using autologous tumor homogenate (as DCVax-L by Liau), instead of allogeneic antigens (DC vaccine by Lepski) could be to target the individual patient’s antigens repertoire, while addressing the extreme heterogeneity of glioblastoma. Our vaccine’s favorable toxicity profile and mechanisms of action make it an ideal candidate for future combination therapies trials.

When used with standard RT-CT treatments, the here proposed autologous DCVax is safe and has met the first step primary endpoint, allowing enrollment to continue.

## Data Availability

The raw data supporting the conclusions of this article will be made available by the authors, without undue reservation.
