# Erratum: First step results from a phase II study of a dendritic cell vaccine in glioblastoma patients (CombiG-vax)

**DOI:** 10.3389/fimmu.2024.1494021

**Published:** 2024-09-19

**Authors:** Frontiers Production Office

**Affiliations:** Frontiers Media SA, Lausanne, Switzerland

**Keywords:** glioblastoma, vaccine, immunotherapy, dendritic cell, adoptive cell therapy, radiochemotherapy

Due to a production error, an author name was incorrectly spelled as “Monia D’Allagata”. The correct spelling is “Monia Dall’Agata”.

The publisher apologizes for this mistake.

The original version of this article has been updated.

