# Supplementary material for: First step results from a phase II study of a dendritic cell vaccine in glioblastoma patients (CombiG-vax)
Source: Front Immunol. 2024 Aug 13;15:1404861. doi: 10.3389/fimmu.2024.1404861 (PMC11347333; doi:10.3389/fimmu.2024.1404861)
Supplement: Supplementary file 1 [file DataSheet1.pdf]

Vaccination with Autologous Dendritic cells loaded with Autologous Tumour homogenate in Glioblastoma: a phase II Study

Eudract number: 2020-003755-15

Protocol Code IRST: IRST191.05

IRST Identifier Code: L2P2308

Date and Version No: 17/02/2022– Amendment 1.0

Acronym: Combi G-Vax

Chief investigator:
Dott.ssa Laura Ridolfi

Sponsor/Promoter:
IRCCS Istituto Romagnolo per lo Studio dei Tumori "Dino Amadori" - IRST S.r.l.
Via Piero Maroncelli, 40/42
47014 Meldola (FC)
Phone +39 0543 739100

Confidentiality Statement
This document contains confidential information that must not be disclosed to anyone other than the Sponsor/Promoter, the Investigator's Team, IRST IRCCS, regulatory authorities, and members of the Ethics Committee.

Protocol Template rev.1_10.07.2020

KEY TRIAL CONTACTS

**Chief investigator**	Dott.ssa Laura Ridolfi<br>U.O. Immunoterapia e Laboratorio TCS<br>IRCCS Istituto Romagnolo per lo Studio dei Tumori "Dino Amadori" - IRST S.r.l.<br>Via Piero Maroncelli, 40/42 - 47014 Meldola (FC)<br>email: laura.ridolfi@irst.emr.it<br>tel. +39 0543 739274 - +39 0543 739927/ fax +39 0543 739221
**Key Protocol Contributors**	Dott. Toni Ibrahim<br>Dott. Massimo Guidoboni<br>Dott. Francesco De Rosa<br>Dott. Sebastiano Calpona<br>Dott.ssa Lorena Gurrieri<br>Dott.ssa Nada Riva<br>Dott.ssa Valentina Fausti<br>Dott. Alberto Bongiovanni<br>Dott.ssa Elena Amadori
**Immunological Lab**	Dott.ssa Jenni Bulgarelli<br>Dott.ssa Marcella Tazzari
**Cell factory qualified person**	Dott. Massimiliano Petrini
**Cell factory Production**	Dott.ssa Anna Maria Granato (Coordinator)<br>Dott.ssa Claudia Piccinini<br>Dott.ssa Monica Stefanelli
**Cell factory Quality Assessment**	Dott.ssa Elena Pancisi (Coordinator)<br>Dott.ssa Valentina Soldati<br>Dott.ssa Silvia Carloni
**Sponsor/Promoter**	IRCCS Istituto Romagnolo per lo Studio dei Tumori "Dino Amadori" - IRST S.r.l.<br>Via Piero Maroncelli, 40/42 - 47014 Meldola (FC)
**Coordinating Center**	Unità di Biostatistica e Sperimentazioni Cliniche<br>IRCCS Istituto Romagnolo per lo Studio dei Tumori "Dino Amadori" - IRST IRCCS<br>Via Piero Maroncelli, 40/42 - 47014 Meldola (FC)<br>Tel. + 39 0544285813 / Fax +39 0544 285330<br>e-mail address: cc.ubsc@irst.emr.it

Statistician

Dott.ssa Flavia Foca
Unità di Biostatistica e Sperimentazioni Cliniche
IRCCS Istituto Romagnolo per lo Studio dei Tumori "Dino Amadori" - IRST S.r.l.
Via Piero Maroncelli, 40/42 - 47014 Meldola (FC)
e-mail: flavia.foca@irst.emr.it

Trials pharmacist

Dott.ssa Laura Crudi
Farmacia Oncologica
IRCCS Istituto Romagnolo per lo Studio dei Tumori "Dino Amadori" – IRST S.r.l.
Via P. Maroncelli, 40 – 47014 Meldola (FC)
Tel. +39 0543/739968
e-mail: laura.crudi@irst.emr.it

Committees

Writing Committee:
Toni Ibrahim - toni.ibrahim@irst.emr.it
Massimo Guidoboni – massimo.guidoboni@irst.emr.it
Francesco De Rosa – francesco.derosa@irst.emr.it
Laura Mercatali – laura.mercatali@irst.emr.it
Jenny Bulgarelli – jenni.bulgarelli@irst.emr.it
Marcella Tazzari – marcella.tazzari@irst.emr.it
Flavia Foca – flavia.foca@irst.emr.it
Bernadette Vertogen – bernadette.vertogen@irst.emr.it
Linda Valmorri – linda.valmorri@irst.emr.it
Monia Dall'Agata – monia.dallagata@irst.emr.it
Dott.ssa Elena Amadori – elena.amadori@irst.emr.it

Emergency contact

Dott.ssa Laura Ridolfi
U.O. Immunoterapia e Laboratorio TCS
IRCCS Istituto Romagnolo per lo Studio dei Tumori "Dino Amadori" - IRST S.r.l.
Via Piero Maroncelli, 40/42 - 47014 Meldola (FC)
e-mail: laura.ridolfi@irst.emr.it
tel. +39 0543 739274 - +39 0543 739927/ fax +39 0543 739221

PROTOCOL SIGNATURE PAGE

Vaccination with Autologous Dendritic cells loaded with Autologous Tumour homogenate in Glioblastoma: a phase II Study
(Combi G-Vax)

EudraCT number: 2020-003755-15

The undersigned agree and confirm that:

The following protocol has been agreed and accepted and the Chief Investigator agrees to conduct the trial in compliance with the approved protocol and will adhere to the principles outlined in the ICH GCP guidelines, REGULATION (EU) No 536/2014, and any subsequent amendments of the clinical trial regulation, Sponsor/Promoter SOP's and other regulatory requirements as amended.

The confidential information contained in this document will not be used for any other purpose other than the evaluation or conduct of the clinical investigation without the prior written consent of the Sponsor/Promoter.

The findings of the study will be made publically available through publication or other dissemination tools without any unnecessary delay and that an honest accurate and transparent account of the study will be given; and any discrepancies from the study as planned in this protocol will be explained.

Laura Ridolfi<br>Chief Investigator	____________________<br>Signature	____________<br>Date
Flavia Foca<br>Trial Statistician	____________________<br>Signature	____________<br>Date

By signing this document, I am confirming that I have read the protocol for the above study and I agree to conduct the study in compliance with the protocol and ICH GCP

____________<br>Principal Investigator	____________________<br>Signature	____________<br>Date

SUMMARY

**Title**	**Vaccination with Autologous Dendritic cells loaded with Autologous Tumour homogenate in Glioblastoma: a phase II Study**
**Short Title/ Acronym**	**Combi G-Vax**
**Protocol Code**	IRST191.05<br>IRST Identifier Code: L2P2308
**Phase**	II
**Study Design**	Single arm, monocentric trial to assess the safety and the progression-free survival related to the combined treatment of dendritic cell vaccine loaded with autologous tumor homogenate and temozolomide in patients operated for glioblastoma and then treated with standard radiochemotherapy (according to Stupp regimen).
**Background and Rationale for the study**	Glioblastoma (GBM) is a poor prognosis malignant WHO grade IV glioma. It is the most common malignant primary central nervous tumor in adults. Standard therapy, after surgical resection, consists of radiotherapy (RT) and chemotherapy (CT) with temozolomide (TMZ), by Stupp et al. However, the prognosis remains poor with a 5-year survival of 5%. Traditional CT has found little success, while TMZ is approved, the majority of tumors are MGMT unmethylated and show a diminished response to this drug. Past decades knew a renovate interest in immunotherapy of cancer due to new drugs and effective therapies like immune-check points inhibitors or adoptive T-cell approaches or dendritic cell-based vaccines or combinations of these. GBM has an immunosuppressive microenvironment due to tumor-associated factors: overexpression of inhibitory cytokines or checkpoint molecules, low level of HLA and elevated infiltrating T-regulatory cells (T-reg). Dendritic cells (DCs) are the most potent professional antigen-presenting cells and due to their function of linkage between innate and adaptive immune response, DCs have become a promising way to generate a specific immune response against various cancers. DC vaccines have been clinically investigated in a vast range of malignancies. Regarding HGGs (High-grade gliomas) multiple phase I/II trials have been reported; however, the objective response rate was only 15.6%. Conversely, two meta-analyses published in 2014 indicated improved survival (OS) and progression-free survival (PFS) with DC vaccination in HGGs patients. Moreover, a most recent meta-analysis confirmed an advantage for DC vaccination in terms of OS and PFS without severe adverse events (AEs) and despite cycles, dosages and rout of administration. Since 2001, we have treated more than 80 advanced melanoma patients with a tumor lysate loaded autologous DC vaccine, obtaining a clinical benefit of 54.1% without meaningful treatment-related toxicity. Patients developing antitumor immunity after vaccination have a better clinical course but only two thirds of patients are immunoresponsive. In these latter patients, DC vaccination strongly increases the amount of intratumoral activated cytotoxic T lymphocytes and decreases the number of FoxP3 positive regulatory T cells, indicating changes conducive to Th1-biased tumor microenvironment. Among the 81 vaccinated patients, seventeen were elderly patients with a mean age of 69 years. Seven out of seventeen patients had metastatic melanoma M1c and 11/17 were pre-treated. We observed 5/17 stable disease and 8 in vivo immunologic response (positive DTH skin test). The

	median OS for the group was of 8.38 months. The toxicity profile was very favorable with no G3-4 side effects correlated to treatment and only one grade 3 and one grade 4 tromboembolism non treatment-related. The DC vaccine produced at IRCCS IRST Cell Factory was shown to induce qualitative and quantitative changes of tumor-associated immune cells indicative of a switch towards a Th1-biased immune environment. Based on these data we propose an immunotherapy the DC vaccine concomitant to standard radiochemotherapy in patients undergone to radically surgery for GBM.	
**Timelines**	Estimated duration for the main protocol: 55 months<br>Enrolment: 30 months<br>Treatment period: 13 months<br>Follow up: 12 months for each patient	
**Study Center(s)**	Single-center: IRCCS Istituto Romagnolo per lo Studio dei Tumori " Dino Amadori"-IRST S.r.l.	
**Objectives**	Primary objectives are clinical activity and safety of the study treatment. Secondary objectives are the evaluation of the prognostic role of the positive skin test DTH after at least four vaccine administrations, the OS and the immunological efficacy of the study treatment. A series of exploratory objectives will be also assessed on samples from patients who participate to this optional part of the trial.	
	Objectives	Outcome measures
**Primary**	To assess clinical activity and safety of the combined study treatment	- Progression free survival (PFS), measured as the proportion of patients without progression of disease at three months from leukapheresis.<br>- Proportion of patients experienced grade 3 or higher adverse events related to the study treatment
**Secondary**	- Immune response *in vivo*<br>- Clinical Outcome<br>- Immunological efficacy	- Evaluation of the prognostic role of a positive DTH test after at least four vaccine administrations<br>- Overall survival (OS)<br>- Ability to enhance the proportion of circulating immune effectors specific for tumor antigens; evaluation of the persistence of an anti-tumor immune response; determination of plasma levels of a panel of inflammatory cytokines and pro-angiogenic factors; evaluation of the prognostic and predictive role of tumor antigen expression in tumor tissue; analysis of the prognostic and predictive role of immune cells in the peripheral blood and in the tumor microenvironment.
**Number of**	28 (9 in the first step, 19 in the second)	

**Subjects**	
**Diagnosis and Main Inclusion Criteria**	During the pre-screening phase, patients underwent the necessary procedures to obtain the biological material for DC vaccine preparation (leukapheresis) and are treated with standard radiochemotherapy (according to Stupp regimen).<br>After pre-screening, patients will be enrolled based on subsequent elegibility criteria:<br>**Inclusion Criteria**<br>1. Histologically confirmed glioblastoma.<br>2. The autologous surgical specimen needed for vaccine manufacturing must have been collected and sent to the Somatic Cell Therapy Lab of IRCCS IRST and must fulfill all the acceptance criteria prescribed by the GMP procedures.<br>3. Availability of sufficient leukapheretic material for the preparation of the vaccine product.<br>4. Patients must have recovered (grade 1 or less by CTCAE 5.0) from all the events related to previous treatments.<br>5. Be willing and able to provide written informed consent/assent for the trial.<br>6. Be ≥ 18 years of age on day of signing informed consent.<br>7. Have a Karnofsky performance status (KPS) ≥ 70% or a performance status of 0 or 1 on the ECOG Performance Scale.<br>8. Demonstrate adequate organ and marrow function.<br><br>**Main Exclusion criteria**<br>1. Patients with a diagnosis of immunodeficiency or receiving systemic steroid therapy > 20 mg prednisone equivalent or any other form of immunosuppressive therapy within 7 days prior to the first dose of trial treatment.<br>2. Patients with active autoimmune disease that has required systemic treatment in the past 2 years (i.e. with use of disease modifying agents, corticosteroids or immunosuppressive drugs). Replacement therapy (eg., thyroxine, insulin, or physiologic corticosteroid replacement therapy for adrenal or pituitary insufficiency, etc.) is not considered a form of systemic treatment.<br>3. Known history of active TB (Bacillus Tuberculosis).<br>4. Previous treatment with a cancer vaccine.<br>5. Other known malignant neoplastic diseases in the patient's medical history with a disease-free interval of less than 5 years, except basal or squamous cell carcinoma of the skin and in situ carcinoma of the cervix uteri treated with radical surgery.<br>6. Any known history of or is positivity of any serologic marker indicative of infection by Treponema pallidum, hepatitis B virus (HBsAg, HBsAb, HBcAB), hepatitis C virus (HCVAb, HCV RNA quantitative), human immunodeficiency virus (HIV), whether actual or previous.<br>7. Patients who have received a live vaccine within 30 days of planned start of study therapy.
**Study Product, Dose, Route, Regimen and duration of administration**	The experimental treatment consists of an induction phase with 4 weekly doses of dendritic cell vaccine ($10x10^6$ cells) intradermally administered (weeks 1-4), followed by a maintenance phase consisting of 28 days cycles with vaccine administration (start on week 7) and adjuvant temozolomide (150-200mg/m^2/day) assumed orally from day 1 to 5 q28 (start on week 5). The combined maintenance treatment will continue until disease progression, unacceptable toxicity or withdrawal of consent

	by the patient, or up to a maximum of 1 year of treatments. After disease progression or the end of maintenance phase, is foreseen a one-year follow-up phase for each subject.<br>If the vaccine runs out, the experimental treatment continues with temozolomide alone.
**Reference therapy**	Stupp regimen
**Statistical Methodology**	Simon's two-stage design (Simon, 1989) will be used for the sample size calculation. A planned interim analysis will be done after the recruitment of the first 9 evaluable patients for toxicity and for efficacy.<br>If study will not be stopped due to lack of safety or efficacy, a total of 28 evaluable patients will be enrolled for the trial.<br>Time to events (PFS and OS) will be calculated with the Kaplan-Meier method and the analysis was performed on the eligible population. For the primary objective, the proportion of patients without progression at three months from leukapheresis date will be evaluated. The proportion of patients experiencing vaccine-related grade ≥ 3 AEs during the treatment will be inferred by means of the two-sided Clopper-Pearson, or a more appropriate one, 95% confidence interval. Descriptive statistics will be used to assess the extent of the secondary endpoints.

ABBREVIATIONS

AE	Adverse event
ALT	Alanine aminotransferase (previously SGPT)
ANC	Absolute neutrophil count
ASAT	Aspartate aminotransferase (previously SGOT)
AR	Adverse reaction
BSA	Body surface area
CBC	Complete blood count
CC	Coordinating centre
CEA	Carcino-embryogenic antigen
CHF	Congestive heart failure
CIC	onfidence interval
CNS	Central Nervous System
CRF	Case Report Form
CRO	Contract Research Organisation
CT	Clinical Trials
CTscan	Computed tomography
CTC	Common toxicity criteria
DSC	Dynamic susceptibility contrast
ECG	Electrocardiogram
ECOG	Performance status (Eastern Cooperative Oncology Group, ECOG Scale)
FPFV	First Patient First Visit
GBM	Glioblastoma
GCP	Good Clinical Practice
G-CSF	Granulocyte colony stimulating factor
GP	General Practitioner
HGG	High grade glioma
HLA	Human leukocyte antigen
IB	Investigators Brochure
ICF	Informed Consent Form
ICH	International Conference of Harmonisation
IDMC	Indipendent Data Monitoring Committee
IEC	Independent Ethics Committee
IMP	Investigational Medicinal Products
IRB	Independent Review Board
LPFV	Last Patient First Visit
LP	Last Patient
LPLV	Last Patient Last Visit
MGMT	O6-methylguanine-DNA-methyltransferase
MHC	Major Histocompatibility Complex
OR	Overall Response Rate
OS	Overall survival

PD	Progressive disease
PFS	Progression Free Survival
PI	Principal Investigator
PIL	Participant/ Patient Information Leaflet
PR	Partial response
RANO	Revised Assessment in Neuro-Oncology
RECIST	Response Evaluation Criteria In Solid Tumors
SAE	Serious Adverse Event
SAP	Statistical Analysis Plan
SAR	Serious Adverse Reaction
SD	Stable disease
SOP	Standard Operating Procedure
SUSAR	Suspected Unexpected Serious Adverse Reactions
TH1	T helper 1
TML	Tumor mutational load
TMZ	Temozolomide
ULN	Upper limit of normal
WBC	White blood cells
WHO	World Health Organization

STUDY SCHEMA

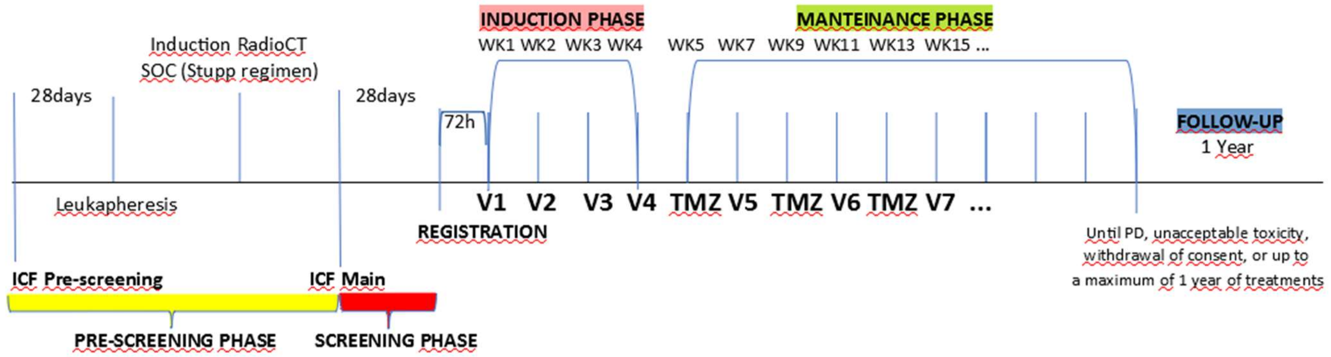

TABLE OF CONTENTS

KEY TRIAL CONTACTS 2
PROTOCOL SIGNATURE PAGE 3
SUMMARY 5
ABBREVIATIONS 9
STUDY SCHEMA 11

1. INTRODUCTION 15
1.1 BACKGROUND 15
1.2 INVESTIGATIONAL AGENT (S) 16
1.3 DELAYED-TYPE HYPERSENSITIVITY SKIN TEST (DTH) 17
1.4 PRECLINICAL DATA 17
1.5 RATIONALE 18

2. OBJECTIVES AND OUTCOME MEASURES/ENDPOINTS 18
2.1. PRIMARY OBJECTIVES 18
2.2 SECONDARY OBJECTIVES 18
2.2.2. SECONDARY IMMUNOLOGICAL OBJECTIVES 19
2.2.3 EXPLORATIVE OBJECTIVES (NOT MANDATORY) 19
2.3 PRIMARY ENDPOINT/OUTCOME 19
2.4 SECONDARY ENDPOINTS/OUTCOMES 19
2.5 SECONDARY IMMUNOLOGICAL AND EXPLORATIVE ENDPOINTS/OUTCOMES 20

3. STUDY DESIGN 21
3.1 SUMMARY OF TRIAL DESIGN 21
3.2 END OF TRIAL DEFINITION 22

4. STUDY POPULATION 22
4.1 INCLUSION CRITERIA FOR THE TRIAL 23
4.2 EXCLUSION CRITERIA FOR THE TRIAL 23

5. STUDY PROCEDURES 24
5.1 PRE-SCREENING AND INFORMED CONSENTS 25
5.2 PRE-SCREENING AND SCREENING PROCEDURES 25
5.3 REGISTRATION PROCEDURE 26
5.4 TREATMENT PHASE 27
5.5 ASSESSMENTS DURING TREATMENT PERIOD 27
5.6 END OF TREATMENT ASSESSMENTS 28
5.8 POST TREATMENT/FOLLOW-UP VISITS 28
5.9 DEFINITIONS 29
5.10 DISCONTINUATION/ WITHDRAWAL OF PARTICIPANTS FROM STUDY TREATMENT 29
5.11 SOURCE DATA 30

6. STUDY TREATMENT 31
6.1 DESCRIPTION OF STUDY TREATMENT 31
6.2 SUPPLY OF STUDY TREATMENT 32
6.2.1. Autologous DC vaccine 32
6.2.1.1. Product description 32
6.2.1.2. Storage requirements 32
6.2.1.3. Stability 32
6.2.1.4 Route of administration 33
6.2.2. Temozolomide 33
6.3 COMPLIANCE WITH STUDY TREATMENT 33
6.4 ACCOUNTABILITY OF THE STUDY TREATMENT 33

6.5 Concomitant Medication ... 33

7. DOSING DELAYS / DOSE MODIFICATIONS ... 34

7.1 Monitoring and Toxicity Management ... 34

8. BIOMARKER / CORRELATIVE / SPECIAL STUDIES (IF APPLICABLE) ... 34

8.1 Materials and methods ... 34
8.3 Samples shipment ... 36
8.4 Sample analysis, confidentiality and sample destruction ... 37

9. MEASUREMENT OF EFFECT ... 37

9.1 Efficacy Parameters ... 37
9.2 Immunological endpoints assessment ... 38
9.3 DTH skin test ... 38
9.4 Method and Timing ... 39

10. SAFETY REPORTING ... 39

10.1 Definitions ... 40
10.2 Reporting Procedures for All Adverse Events ... 42
10.3 Reporting Procedures for Serious Adverse Events ... 43

11. STATISTICAL CONSIDERATIONS ... 44

11.1 Study Design/Endpoints ... 44
11.2 Sample Size, Accrual Rate and Study Duration ... 45
11.3 Stratification Factors ... 45
11.4 Analysis of Primary Endpoints ... 45
11.5 Analysis of Secondary Endpoints ... 46
11.6 Interim Analysis and Criteria for termination of the trial ... 46
11.7 Reporting and Exclusions ... 46
11.8 Procedure(s) to account for missing or spurious data ... 46
11.9 Other statistical considerations ... 47

12. ETHICAL ASPECTS ... 47

12.1 Declaration of Helsinki ... 47
12.2 ICH Guidelines for Good Clinical Practice ... 47
12.3 Independent Ethical Committee (IEC) ... 47
12.4 Informed Consent ... 47
12.5 Patient data protection ... 48

13. DATA COLLECTION ... 48

14. STUDY MONITORING ... 49

14.1 Site Set-up and Initiation ... 49
14.2 On-site Monitoring ... 49
14.3 Central Monitoring ... 50
14.4 Audit and Inspection ... 50

15. ADMINISTRATIVE REGULATIONS ... 50

15.1 Curriculum vitae ... 51
15.2 Secrecy agreement ... 51
15.3 Availability and Retention of Investigational Records ... 51
15.4 Insurance ... 51

16. OWNERSHIP OF DATA AND USE OF THE STUDY RESULTS 52
17. PUBLICATION POLICY 52
18. PROTOCOL AMENDMENTS 52
19. REFERENCES 54
APPENDIX A PERFORMANCE STATUS CRITERIA 56
APPENDIX B NCI COMMON TERMINOLOGY CRITERIA FOR AE 57
APPENDIX C SCHEDULE OF PROCEDURES 58
APPENDIX D RESPONSE CRITERIA 60
APPENDIX E WORLD MEDICAL ASSOCIATION DECLARATION OF HELSINKI 61

1. INTRODUCTION

This document is a protocol for a human research study. This clinical trial is to be conducted in compliance with the protocol, with the REGULATION (EU) No 536/2014, with the principles of ICH Good Clinical Practice, and institutional research policies and procedures.

1.1 Background

Glioblastoma (GBM) is a poor prognosis malignant WHO grade IV glioma. It is the most common malignant primary central nervous tumor in adults. Standard therapy, after surgical resection, consists of radiotherapy (RT) and chemotherapy (CT) with temozolomide (TMZ), by Stupp et al. However, the prognosis remains poor with a 5-year survival of 5%[1].

Traditional CT has found little success, while TMZ is approved, the majority of tumors are MGMT unmethylated and show a diminished response to this drug. The failure of current therapy to adequately treat GBM has prompted clinicians to look for novel approaches minimizing harm to health cells. [2-3]

Past decades knew a renewed interest in immunotherapy of cancer due to new drugs and effective therapies like immune-checkpoints inhibitors or adoptive T-cell approaches or dendritic cell-based vaccines or combinations of these. GBM has an immunosuppressive microenvironment due to tumor-associated factors: overexpression of inhibitory cytokines or checkpoint molecules, low expression levels of HLA molecules and elevated levels of infiltrating regulatory T cells (T-reg). [4-5]

Dendritic cells (DCs) are the most potent professional antigen presenting cells that express both MHC 1 and 2 molecules and are the most efficient stimulus of new T- and B-cell responses. Due to their function of linkage between innate and adaptive immune response, DCs have became a promising way to generate a specific immune response against various cancers. [6-8]. DC vaccines have been clinically investigated in a vast range of malignancies including prostate cancer, melanoma, renal cell carcinoma and even glioma [9]. Regarding HGGs (High-grade gliomas) multiple phase I/II trials have been reported; close to 500 patients with GBM have been treated with DC vaccination in more than 38 studies and all of these documented feasibility and safety. [10-12] Even if the objective response rate was only 15.6% two meta-analysis published in 2014 [13] and some controlled studies indicated improved survival (OS) and progression free survival (PFS) with DC vaccination in HGGs patients.[14] In 16 non-randomized studies the median OS of newly diagnosed GBM patients ranged from 11.0 to 38.4 months. Moreover, a systematic review by Wang X. of 171 studies confirmed an advantage for DC vaccination in terms of OS and PFS without severe adverse events (Ads) and despite of cycles, doses and route of administration.[15]

GBM is configured as a typical “immune-deserted” cancer exhibiting a number of systemic and environmental immunosuppressive factors, a scarce immune infiltrate characterized by a paucity of T-cells, a massive recruitment of immunosuppressive cells, a low tumor mutational load (TML) with a consequent low neoantigen burden and low immunogenicity [16-17].

Since 2001, we have treated more than 80 advanced melanoma patients with a tumor lysate loaded autologous DC vaccine, obtaining a clinical benefit of 54.1% without meaningful treatment-related

toxicity. Patients developing antitumor immunity after vaccination have a better clinical course, but only two thirds of patients are immune responsive[18]. In these latter patients, DC vaccination induced a significant increase of CD8+ TILs and in general exerts an important role in sustaining or de novo inducing a T cell inflamed TME. [19]. The toxicity profile was very favorable with no grade 3-4 side effects correlated to treatment and only one grade 3 and one grade 4 tromboembolism not treatment-related. In addition, preliminary data obtained in the PD-L1 negative subset of this series indicates that the treatment induces PD-L1 expression in tumor cells in almost all cases.

DC vaccination can be easily integrated into first-line therapy and there is a rational for thus integration:

- After resection/radio-chemotherapy patients are in a state of minimal residual disease which is probably beneficial for immunotherapy because of the lower tumor load and depletion of immunosuppressive cells
- TMZ may reduce regulatory T cell
- The lymphocyte compartment recovering after chemotherapy appears to be beneficial for the induction of anti-tumor responses
- Dying tumor cells after radio-chemotherapy may act as danger signal and boost an effective antitumor immune response
- There is an increased responsiveness to TMZ after DC vaccination

1.2 Investigational agent (s)

Since 2001, IRST Somatic Cell Therapy Lab (Cell Factory) is producing an advanced medicinal consisting of a therapeutic vaccine constituted by autologous DC pulsed with autologous tumor lysate or homogenate in patients with metastatic melanoma or kidney cancer (Clinical trial N° 800/II A.48.3/2099 “Vaccinazione con cellule dendritiche in pazienti affetti da melanoma”).

This vaccine is now produced according GMP procedures in the IRST Cell factory, which was authorized by AIFA for cell therapy production.

The “autologous dendritic cell loaded with autologous tumor homogenate” is an Advanced Therapy Medicinal Product consisting of dendritic cells obtained by in vitro differentiation of peripheral blood monocytes, isolated by leukapheresis from each patient, with IL-4 and GM-CSF. Immature DC such obtained are then loaded with a homogenate of tumor tissue obtained from the same patient, matured with a cytokine cocktail containing IL1b, PGE2, IL6, and TNF-a (“maturation cocktail”). Pulsed mature dendritic cells (mDC) are collected at day 9, washed, counted, tested for quality control (vitality, purity, phenotype markers, sterility, endotoxin, mycoplasma) frozen in aliquots (at least 13×10^6 cells/aliquot) and stored in nitrogen vapours. The aliquots are thawed and packed in two insulin syringes for administration to the patient ($10x10^6$ total dendritic cells). The syringes are filled and closed in Class A area and report the product identity. The syringes are packed in their original packaging labeled and put in a plastic bag reporting the product and the protocol identity, according to EU-cGMP Annex XIII.

The content of each syringe is administered intradermally with 5 injections in sites close to inguinal or axillary lymph node stations that had not been site of previous surgical exeresis, preferentially alternating injection sites in consecutive vaccine administrations.

1.3 Delayed-type hypersensitivity skin test (DTH)

DTH testing is a classical method for measuring cell-mediated immune reactivity. This technique involves intradermal administration of antigen preparation and recording the degree of erythema and induration produced 24-48 hours after the injection. This response reflects antigen-specific recruitment and activation of CD4+ to release T helper-1 cytokines (IFN-γ in particular) and recruitment and activation of CD8+ effector T cells in the injection site (18). In our and others' groups experience, positive response to DTH test performed with soluble antigen after vaccination with DC vaccine in patients carrying metastatic melanoma was strongly related with better clinical outcome. In addition, DTH testing does not require extensive training nor the utilization of costly equipment, and can be easily performed at the bedside. All these features make it a feasible, low-cost immunomonitoring method to evaluate immunologic efficacy in a clinical trial setting.
DTH testing dose corresponds to 50 µg of autologous tumor homogenate or KLH (positive control) prepared in 0.5 ml of 0.9% sterile saline. The negative control is a dose of 0.5 ml of 0.9% sterile saline alone.

1.4 Preclinical Data

Recently, the Immunotherapy and Somatic cell therapy Unit (IRST) have published a study in which quantitative and qualitative changes in tumor-infiltrating T lymphocytes (TILs) induced by vaccination with autologous tumor lysate/homogenate loaded DCs have been investigated in a series of 16 patients with metastatic melanoma. Immunohistochemistry for CD4, CD8, Foxp3, Granzyme B (GZMB), PDL1, and HLA class I was performed in tumor biopsies collected before and after DC vaccination. The density of each marker was quantified by automated digital pathology analysis on whole slide images. Co-expression of markers defining functional phenotypes, i.e., Foxp3+ regulatory CD4+ T cells (Treg) and GZMB+ cytotoxic CD8+T cells, was assessed with sequential immunohistochemistry. A significant increase of CD8+ TILs was found in post-vaccine biopsies of patients who were not previously treated with immune-modulating cytokines or Ipilimumab. Interestingly, along with a maintained tumoral HLA class I expression, after DC vaccination we observed a significant increase of PDL1+ tumor cells, which significantly correlated with intratumoral CD8+ T cell density. This observation might explain the lack of a significant concurrent cytotoxic reactivation of CD8+ T cell, as measured by the numbers of GZMB+ T cells. Altogether our findings indicate that DC vaccination exerts an important role in intratumoral T cell activation detected in post-DC therapy lesions, that is lessened by an occurring phenomenon of adaptive immune resistance, yet the concomitant PDL1 up-regulation [19].

Numerous preclinical studies have attempted to evaluate the efficacy and feasibility of DC vaccine in gliomas. One of the earliest studies of glioma immunization attempted to demonstrate that therapeutic immunization in established tumors is possible. Siesjo et al. showed that pre-immunization of mutagen-treated rat glioma N32 cells led to the rejection of subsequent subcutaneous injection and intracerebral implantation of weakly immunogenic unmutated N32 gliomas. The group subsequently demonstrated that immunization of weakly immunogenic unmutated tumor cells with adjuvants such as DCs led to

significant therapeutic effects equivalent to the clinical benefits of immunization with mutated cell lines [20]. A similar experimental model using the 9L rat glioma cell line yielded similar results and showed the effectiveness of DCVs in cytotoxic CD8+ T cell-mediated anti-tumor immunity [21]. The authors demonstrated increased infiltration of CD8+ T cells in the TME as shown by immunohistochemistry (IHC) as well as increased in vitro 9L cell lysis by CTLs after vaccine treatment compared to the control group. Despite differences in techniques, these studies demonstrated the potential of DC vaccines to elicit anti-tumor response.

1.5 Rationale

Since 2001, we have treated more than 80 advanced melanoma patients with a tumor lysate loaded autologous DC vaccine, obtaining a clinical benefit of 54.1%. The results show that, in patients who develop an immune response directed to the vaccination antigens (about two thirds), OS is significantly improved in comparison both to the other patients and to advanced melanoma patients treated with chemotherapy [18]. Another study evaluated DC vaccine in combination with low-dose temozolomide aimed at reducing the number of T regulatory cells [22], and another clinical trial is ongoing. All the studies confirm the good safety profile of the vaccine, similarly to other studies.

DC vaccination can be easily integrated into first-line therapy and there is a rationale for this integration:

- After resection/radio-chemotherapy patients are in a state of minimal residual disease which is probably beneficial for immunotherapy because of the lower tumor load and depletion of immunosuppressive cells
- TMZ may reduce regulatory T cell
- The lymphocyte compartment recovering after chemotherapy appears to be beneficial for induction of anti-tumor responses
- Dying tumor cells after radio-chemotherapy may act as danger signal and boost an effective antitumor immune response
- There is an increased responsiveness to TMZ after DC vaccination

2. OBJECTIVES AND OUTCOME MEASURES/ENDPOINTS

2.1. Primary objectives

Progression-free survival (PFS) will be evaluated, intended as the proportion of patients without progression of disease at three months from leukapheresis date. Evaluation of safety will be considered as co-primary endpoint.

2.2 Secondary objectives

1. To evaluate the prognostic role of a positive DTH test after at least four vaccine administrations.
2. To assess overall survival (OS)

2.2.2. Secondary Immunological objectives

3. To determine the immunological efficacy of the vaccine in terms of its ability to enhance the proportion of circulating immune effectors specific for tumor antigens known to be expressed in these tumors by INF-γ ELISPOT assay.
4. To evaluate the persistence of an anti-tumor immune response after the completion of the vaccination program both in relapsed and in disease-free patients
5. To measure the plasma levels of a panel of inflammatory cytokines and pro-angiogenic factors in longitudinal plasma samples by cytometric bead array.
6. To evaluate the prognostic and predictive role of tumor antigen expression in tumor tissue by immunohistochemistry (IHC).
7. To define the prognostic and predictive role of immune cells in the peripheral blood as well as in the tumor microenvironment (TME).

2.2.3 Explorative objectives (NOT MANDATORY)

As an explorative objective of this study the HLA class I and II characterization of patients will be assessed. The typing of the locus A-B-C-DR and DQA/DQB will be performed by the Genetic Laboratory of by means of the sequence-specific oligonucleotide probe reverse hybridization (PCR-SSO) method. This information could be useful to designed personalized functional in vitro immune response assays, indeed in the IFN-γ ELISPOT assay lymphocytes could be stimulated with HLA-class I restricted peptides to better characterized the specific and personalized immunological response of each patients to target antigens. As further point we will evaluate whether certain HLA aplotype could have a correlation with the patients clinical outcome.

Patients will check in the informed consent form if they will participate to this facultative part of the trial.

2.3 Primary endpoint/outcome

- Progression free survival (PFS) is the time from the date of leukapheresis to the date of first progression or the date of death from any cause or the date of the last restaging in non-progressed patients. For the evaluation of primary objective, the proportion of patients without progression of disease at three months from the date of leukapheresis will be calculated.

- The co-primary endpoint of the study will be the safety of the study treatment (see section 6). The adverse events recorded during the observation period (i.e. from the day of the first DC vaccine dose administered in induction phase up to 30 days after the last dose of study treatment), will be reported and graded according to NCI CTCAE 5.0, their severity and causal relationship with the investigational product will be analyzed by means of descriptive statistics.

2.4 Secondary endpoints/outcomes

- *In vivo* Immunomonitoring, will be measured by DTH test against tumor homogenate and KLH after

at least the 4 vaccine doses (induction). DTH testing will be performed in all patients on day 0 (pre-treatment DTH, which showed no reactivity in the large majority of patients so far evaluated in our previous studies), after induction phase (post-treatment DTH), every 2 cycle of maintenance phase and at the end of treatment. The diameter of induration/erythema observed after 24 hrs is recorded according to the following scale: 0-5 mm grade 1, 6-10 mm grade 2, 11-20 mm grade 3, > 21 mm grade 4. As DTH reactivity to lower concentrations of the antigen(s) is strictly related to more intense antigen specific immune responses, score results will be normalized against the concentration itself and transformed into a 0-80 scale for analysis purposes. Best result obtained for each patient at any of the post-treatment DTHs, either for ATL or KLH, will be taken into account for data analysis (best normalized score). Patients DTH scores will be also evaluated in combination with IFN-γ-ELISPOT analysis of circulating effectors.

- The OS will be measured from the date of leukapheresis until the date of death from any cause or the last date on which it was known that the patient was alive.

2.5 SECONDARY IMMUNOLOGICAL AND EXPLORATIVE ENDPOINTS/OUTCOMES

1. The immunological efficacy will be assessed as a proportion of tumor-specific circulating immune effectors determined by IFN-γ ELISPOT assay at baseline and in concomitance with subsequent disease restaging per protocol (at 4 months, after at least 3 vaccinations) after stimulation with peptide libraries fully covering the sequences of a panel of tumor-associated antigens (TAAs). Moreover, accordingly with patient’s HLA haplotype (where available) lymphocytes will be stimulated with HLA- restricted peptides to better characterized the specific and personalized immunological response of each patients. Enhancement of at least 10% of antigen-specific spot-forming cells (SFC) subtracting spots obtained with unstimulated PBMC, for at least one antigen after treatment compared with baseline sample, will be considered as positive.
2. The assessment of the persistence of anti-tumor specific effector cells, will be assessed by IFN-γ ELISPOT assay, after the completion of the vaccination program both in relapsed and in disease-free patients.
3. The plasma levels of pro-inflammatory/pro-angiogenic factors (such as IL-1beta, IL-6, IL-8, IL-10, IL-12p70, TNFalpha, VEGF and Fibronectin) will be assessed in longitudinal plasma samples by cytometric bead array. The results will be analysed to evaluate whether specific marker expression profiles at baseline, and/or changes induced by vaccinations are able to predict the clinical outcome.
4. For the assessment of the prognostic and predictive role of tumor antigen expression in tumor tissue, IHC analyses will be conducted on FFPE samples to evaluate the expression of TAAs (such as SOX2, SOX11, EZH2, TNC, BIRC5/SURVIVIN, IL13Ra2, HER2, IGF2BP3, MAGEA1, MAGEA3, GP100). The results will be analysed to evaluate whether the expression of these TAAs is able to predict the clinical outcome.
5. The functional phenotyping of circulating and in situ immune effectors/regulators will be conducted with the aim to define the predictive role of immune cells in the peripheral blood and in the tumour microenvironment (TME). For the peripheral/systemic analysis, PBMC samples

taken before treatment, after third vaccination and at the end of treatment will be evaluated by multi-parametric flow cytometry for CD3, CD4, CD8, CCR7 and CD45RA to identify the relative proportion of T naïve, T central memory (TCM), T effector memory (TEM), and T effector (TE) cells. Moreover, the proportion of CD14+HLADRlow/neg monocytic myeloid-derived suppressor cells (MDSCs) and FOXP3+ regulatory T cells (Tregs), will be also evaluated by flow cytometry. For the *in situ* analysis, IHC analyses will be performed on formalin-fixed paraffin-embedded (FFPE) samples to identify main immune cell subsets, including T lymphocytes MDSCs and Tregs at the level of the TME. The following markers will be assessed: CD3, CD4, CD8, CD45RO, FOXP3, Granzyme B, TIA-1, CD11b, CD11c, CD14, CD163, HLA-class I, HLA-Class II. To limit the number of FFPE tissue sections as well as to: i) be able to assess complex phenotypes based upon multiple marker co- expression profiles ii) obtain information about reciprocal relation among cell types (e.g. intratumoral proximity) a sequential multiplex IHC protocol developed within the Immunotherapy-Cell Therapy and Biobank Unit will be carried out to perform the above mentioned stainings. Data obtained at both the systemic and in situ levels will be analyzed to evaluate changes in the frequency of these immune subsets upon DC vaccination. A correlation with clinical outcome will also be performed to verify whether specific immune cell subtypes are predictive of efficacy.

3. STUDY DESIGN

3.1 Summary of Trial Design

This is a single-arm, monocentric, phase II study, to assess the safety and the progression-free survival related to the combined treatment of dendritic cell vaccine loaded with autologous tumor homogenate and temozolomide in patients operated for glioblastoma and then treated with standard radiochemotherapy (according to Stupp regimen).
The experimental treatment consists of an induction phase with 4 weekly doses (weeks 1-4) of dendritic cell vaccine (10x106 cells) intradermally administered, followed by a maintenance phase consisting of 28 days cycles with vaccine administration (start week 7, max 1 year of treatment) and adjuvant temozolomide (150-200mg/m2/day) assumed orally from day 1 to 5 q28 (start week 5). The combined maintenance treatment will continue until disease progression, unacceptable toxicity due to vaccine treatment or withdrawal of consent by the patient, or up to a maximum of 1 year of treatment. If the vaccine runs out, the experimental treatment continues with temozolomide alone.
To avoid unacceptable toxicity, a formal safety analysis will be conducted after nine evaluable patients have been observed for at least 30 days after the induction phase (four DC vaccine administration). If three or more patients have experienced grade 3 or higher adverse events related to study treatment, enrollment will be definitively stopped; differently, the other patients to reach the complete study recruitment, will be enrolled.

3.2 End of trial definition

The end of the study is defined as the last study assessment for the last subject on study or anytime the promoter terminates the study, whichever comes first. The coordinating center or Promoter will notify the IEC(s) that the trial has ended and a summary of the clinical trial report will be provided within 12 months of the end of trial.

4. STUDY POPULATION

The study will be conducted on patients with glioblastoma, surgically operated, with ≤ 5 ml residual tumor volume.

After signing the informed consent form for pre-screening (see separated document), a pre-screening phase will start and patient will assess the procedures to obtain sufficient leukapheretic material for the dendritic cell vaccine manufacturing and will perform the standard radiochemotherapy treatment (Stupp regimen) for the disease.

Patient with availability of sufficient leukapheretic material for DC vaccine manufacturing, who have recovered (grade 1 or less by CTCAE 5.0) from all the adverse events related to previous treatments and without disease progression will be candidate to be enrolled in the clinical trial (after signing the study informed consent form, see separated document) if the other eligibility criteria are met.

Patients must have baseline evaluations performed 28 days prior to the registration date and must meet all inclusion and exclusion criteria. In addition, the patients must be thoroughly informed about all aspects of the study, including the study visit schedule, required evaluations and all regulatory requirements for informed consent. The written informed consent must be obtained from the patient prior to any study procedure.

For the pre-screening phase of the study the eligibility criteria are:

1. Histologically confirmed glioblastoma
2. Near-complete resection) confirmed by “central neuroradiologist on magnetic resonance imaging (MRI) or CT scan within 72 h postoperative”
3. Karnofsky performance status (KPS) ≥ 70% or performance status of 0 or 1 on the ECOG Performance Scale (Appendix A)
4. Be willing and able to provide written informed consent/assent for the pre-screening phase of the trial.
5. Be ≥ 18 years of age on day of signing informed consent.
6. Life expectancy of greater than 12 weeks.

7. Patient suitable for the collection of biological material from leukapheresis: serological tests HIV, HBV, HCV, Treponema pallidum negative; normal cardiological parameters (ECG and cardiological examination); evaluation by transfusionist to exclude possible contraindications to leukapheresis.
8. Patient candidate to standard radiochemotherapy (Stupp regimen)
9. Appropriate 12-lead ECG and echocardiogram.

4.1 Inclusion Criteria for the trial

1. Histologically confirmed glioblastoma
2. Patients must have recovered (grade 1 or less by CTCAE 5.0, see appendix B) from all the events related to previous treatments
3. The autologous surgical specimen needed for vaccine manufacturing must have been collected and sent to the Somatic Cell Therapy Lab of IRST IRCCS and must fulfil all the acceptance criteria prescribed by the GMP procedures. (sterile tumor sample of ≥ 150 mg with tumor cell frequency ≥ 60% as determined by central neuropathologist available for vaccine production)
4. Availability of sufficient leukapheretic material for the preparation of the vaccine product.
5. Karnofsky performance status (KPS) ≥ 70% or performance status of 0 or 1 on the ECOG Performance Scale
6. Be willing and able to provide written informed consent/assent for the trial.
7. Be ≥ 18 years of age on day of signing informed consent.
8. Life expectancy of greater than 12 weeks.
9. Patients must have normal organ and marrow function as defined below:
   - Leukocytes >3,000/µL
   - absolute neutrophil count >1,500/µL
   - platelets >100,000/µL
   - total bilirubin within normal institutional limits
   - AST(SGOT)/ALT(SGPT) <2.5 X institutional upper limit of normal
   - creatinine within normal institutional limits
   - OR
   - creatinine clearance >60 mL/min/1.73 m2 for patients with creatinine levels above institutional normal limits
10. Female participants of child bearing potential and male participants whose partner is of childbearing potential must be willing to ensure that they or their partner use effective contraception during the study and for 4 months thereafter.

4.2 Exclusion Criteria for the trial

The participant may not enter the study if ANY of the following apply:

1. Patient is currently participating and receiving study therapy or has participated in a study of an investigational agent and received study therapy or used an investigational device within 4 weeks of the first dose of treatment.

2. Patient with a diagnosis of immunodeficiency or is receiving systemic steroid therapy > 20 mg prednisone equivalent or any other form of immunosuppressive therapy within 7 days prior to the first dose of trial treatment.
3. Medical history of severe acute or chronic disease with poor prognosis, autoimmune disorder, immunodeficiency or organ allograft.
4. Known history of active TB (Bacillus Tuberculosis).
5. Previous treatment with a cancer vaccine.
6. Known allergy or intolerability to components of vaccine, to TMZ.
7. Severe myelosuppression.
8. History of bleeding diathesis or coagulopathy
9. O6-methylguanine-DNA-methyltransferase (MGMT) promoter methylation status equivocal.
10. Other known malignant neoplastic diseases in the patient's medical history with a disease-free interval of less than 5 years, except basal or squamous cell carcinoma of the skin and in situ carcinoma of the cervix uteri treated with radical surgery.
11. Has active autoimmune disease that has required systemic treatment in the past 2 years (i.e. with use of disease modifying agents, corticosteroids or immunosuppressive drugs). Replacement therapy (eg., thyroxine, insulin, or physiologic corticosteroid replacement therapy for adrenal or pituitary insufficiency, etc.) is not considered a form of systemic treatment.
12. Has known history of, or any evidence of active, non-infectious pneumonitis or interstitial lung disease.
13. Has an active infection requiring systemic therapy.
14. Has a history or current evidence of any condition, therapy, or laboratory abnormality that might confound the results of the trial, interfere with the subject's participation for the full duration of the trial, or is not in the best interest of the subject to participate, in the opinion of the treating investigator.
15. Has known psychiatric or substance abuse disorders that would interfere with cooperation with the requirements of the trial.
16. Any known history of or is positivity of any serologic marker indicative of infection by Treponema pallidum, hepatitis B virus (HBsAg, HBsAb, HBcAB), hepatitis C virus (HCVAb, HCV RNA quantitative), human immunodeficiency virus (HIV), whether actual or previous. The sole positivity for antibodies against the HBsAg (i.e. with all other HBV markers negative) is indicative of previous HBV vaccination and therefore is acceptable.
17. Has received a live vaccine within 30 days of planned start of study therapy. (Note: seasonal influenza vaccines for injection are generally inactivated flu vaccines and are allowed; however intranasal influenza vaccines (e.g., Flu-Mist®) are live attenuated vaccines, and are not allowed).

5. STUDY PROCEDURES

All on-study visit procedures are allowed a window of ± 7 days unless otherwise noted. Treatment or visit

delays for public holidays or weather conditions do not constitute a protocol violation.
See Appendix C for schedule of procedures.

5.1 Pre-screening and Informed Consents

The participant must personally sign and date the latest IEC-approved version of the pre-screening ICF and, only patients with availability of sufficient leukapheretic material for DC vaccine preparation, who have recovered (grade 1 or less by CTCAE 5.0) from all the adverse events related to previous treatments and without disease progression must personally sign and date the latest IEC-approved version of the ICF within 28 days before patient registration, before any study-specific procedure is performed.

Written and verbal versions of the participant information and Informed consent will be presented to the participants detailing no less than: the exact nature of the study; the implications and constraints of the protocol; the known side effects and any risks involved in taking part. It will be clearly stated that the participant is free to withdraw from the study at any time for any reason without prejudice to future care, and with no obligation to give the reason for withdrawal.

The participant will be allowed as much time as wished to consider the information, and the opportunity to question the Investigator, their GP or other independent parties to decide whether they will participate in the study. Written Informed Consent will then be obtained by means of participant dated signature and dated signature of the person who presented and obtained the informed consent. The person who obtained the consent must be suitably qualified and experienced, and have been authorised to do so by the Chief/Principal Investigator. A copy of the signed Informed Consent will be given to the participants. The original signed form will be retained at the study site.

5.2 Pre-screening and Screening Procedures

After signing the Informed Consent Form for the pre-screening, a pre-screening phase will start and patient will assess the leukapheresis (within 28 days from pre-screening informed consent signature) and the standard radiochemotherapy treatment (Stupp regimen) as for clinical practice.

Specific Pre-screening procedures are:

- Post-operative brain MRI or brain CT scan.
- Serological markers (Treponema pallidum, Hepatitis B virus (HBsAg, HBsAb, HBcAb), hepatitis C virus (HCVAb, HCV RNA quantitative), Human immunodeficiency virus (HIV)
- Cardiologic evaluation with 12-lead ECG and echocardiogram

If the leukapheretic material collected will not be sufficient for DC vaccine manufacturing or patient will not recover (grade 1 or less by CTCAE 5.0) from all the adverse events related to standard radiochemotherapy (Stupp regimen), patient is not eligible for Combi G-Vax trial.

Patients with availability of sufficient leukapheretic material for DC vaccine manufacturing (Cell Factory of

IRST IRCCS), who have recovered (grade 1 or less by CTCAE 5.0) from all the adverse events related to previous treatments will be candidate to be enrolled in the clinical trial.

After signing the Informed Consent Form for the clinical trial, the screening phase will start and the following evaluations must be performed and completed within 28 days since the registration date.

- Demographics: date of birth, gender, race, smoking and drinking habits
- Complete medical history: details of any history of disease or surgical interventions in the following systems will be recorded.
- History of prior treatments and any residual toxicity relating to prior treatment.
- Recording of Concomitant Medications (see section 6.5).
- Physical Examination: Performance status (ECOG or Karnofsky), height and weight and vital signs: resting pulse and blood pressure (BP) measurements will be measured after the participant has sat for at least five minutes, body temperature will be recorded.
- Laboratory Tests: haemoglobin, haematocrit, platelet count, white blood cell count with absolute neutrophil count and absolute lymphocyte count, glucose, creatinine, creatinine clearance, urea, bilirubin, AST, ALT, alkaline phosphatase, γGT, sodium, potassium, calcium, chloride, magnesium, total protein, albumin.
- Coagulation assessment, including prothrombin time, partial thromboplastin time, international normalized ratio (PT/PTT/INR).
- Pregnancy test (only for women of childbearing potential). A serum pregnancy test is strongly encouraged; however, urine pregnancy test is also acceptable.
- Cardiologic evaluation with 12-lead ECG and echocardiogram, if clinically indicated.
- DTH and blood sampling for secondary and translational endpoints (blood samples and paraffin embedded tissue sections from the primary/metastatic tumor sample).
- HLA blood sample (optional) for exploratory endpoint.
- The laboratory exams (Laboratory Tests, Coagulation assessments and Pregnancy test) should be performed within 10 days from registration date. The other procedures have to be performed within 28 days from registration date.
- All laboratory results will be reviewed and the reports signed by the Investigator who will record in the CRF whether they are normal, abnormal but not clinically significant, or abnormal AND clinically significant. In the last case the eligibility of the participants will be reviewed.

5.3 Registration procedure

Upon completion of the screening the Principal Investigator or a trained delegate will review the medical record of the patient and the results of the screening tests and verify the eligibility of the patient as per inclusion and exclusion criteria (see sections 4.1 and 4.2).
All patients for whom eligibility criteria have been verified will be registered by the Biostatistics and Clinical Trial Unit of IRST (Coordinating Centre, CC). All patients must be registered following the standard IRST procedure prior to initiation of study therapy. For any information regarding study procedures please

contact:

Centro di Coordinamento Studi IRST IRCCS
Unità di Biostatistica e Sperimentazioni Cliniche
IRCCS Istituto Romagnolo per lo Studio dei Tumori "Dino Amadori" – IRST S.r.l.
c/o Ospedale S. Maria delle Croci
Viale Randi, 5 – 48121 RAVENNA (RA)
TEL +39 0544 28 5813 / 5075 -
cc.ubsc@irst.emr.it

Study treatment should be administered within 72 hours of registration.

Further detailed information will be sent to participating centers and will also be included in the Investigator Site File.
Patient accrual rates will be constantly monitored and action will be taken when necessary to resolve recruitment problems.

5.4 Treatment phase

The experimental treatment consists of an induction phase with 4 weekly doses of dendritic cell vaccine (10x106 cells) intradermally administered (weeks 1-4), followed by a maintenance phase consisting of 28 days cycles with vaccine administration (start week 7) and temozolomide (150-200mg/m2/day) assumed orally from day 1 to 5 q28 (start week 5). The combined maintenance treatment will continue until disease progression, unacceptable toxicity or withdrawal of consent by the patient, or up to a maximum of 1 year of treatment. If the vaccine runs out, the experimental treatment continues with temozolomide alone.
After the end of maintenance phase, is foreseen a one-year follow-up phase for each subject.

5.5 Assessments during treatment period

During each visit of treatment phase, the following evaluations should be performed within 3 days before study treatment administration:

- Performance status (ECOG or Karnofsky).
- Physical examination, weight and vital signs (body temperature, pulse and blood pressure).
- Recording of Concomitant Medication.
- Adverse events assessment.
- Laboratory tests: haemoglobin, haematocrit, platelet count, white blood cell count with absolute neutrophil count and absolute lymphocyte count, glucose, creatinine, urea, bilirubin, AST, ALT, alkaline phosphatase, γGT, sodium, potassium, calcium, chloride, magnesium, total protein, albumin.
- Pregnancy test (only for women of childbearing potential).

- Tumour assessment: brain MRI (RANO CRITERIA) after induction phase and every 2 cycles of study treatment during maintenance phase. If there is a questionable pseudoprogression report, the PI or sub-investigators may decide to continue study treatment pending confirmation of progression with subsequent timepoint MRIs if there are no signs and symptoms indicating clear disease progression.The neuroradiologist will distinguish between real progression of GBM from radionecrosis and pseudo progression by evaluating the 5 ROIs of the perfusion study and correlate them with RANO imaging criteria.
- DTH test
- Blood sampling for secondary and translational endpoints (after induction phase and every 2 cycles of study treatment during maintenance phase).

If needed, additional leukapheresis may be planned and performed in a timely manner to ensure the respect of the interval between cycles as specified by the protocol (i.e. 28±7 days). In this case the patient should be evaluated by the Transfusion Medicine specialist and repeat a cardiologic evaluation with 12-lead ECG and echocardiography. Both evaluations should be performed in the 28 days preceding the procedure. The tests for the serologic markers of HBV (including at least anti-HBs antibodies and anti-HBc antibodies), HCV, HIV and Treponema pallidum should also be repeated.

5.6 End of treatment assessments

- The end of treatment visit should be performed 28±7 days after the last treatment cycle or treatment interruption for any reason. The following evaluations should be performed:
- Clinical assessment: performance status (ECOG or Karnofsky).
- Physical examination, weight and vital signs (body temperature, pulse and blood pressure).
- Recording of Concomitant Medication
- Adverse events assessment
- Laboratory tests: haemoglobin, haematocrit, platelet count, white blood cell count with absolute neutrophil count and absolute lymphocyte count, glucose, creatinine, urea, bilirubin, AST, ALT, alkaline phosphatase, γGT, sodium, potassium, calcium, chloride, magnesium, total protein, albumin.
- Tumour assessment: brain MRI, if not performed at the previous treatment cycle.
- DTH test and blood sampling for secondary and translational endpoints, if not performed at the previous treatment cycle.
- In case of treatment interruption because of disease relapse, no further follow-up visit is required, except survival contacts every two months until death or end of study. If indicated, follow-up of adverse events related to the study treatment must be performed.

5.8 Post treatment/follow-up visits

The follow-up phase starts after the end of treatment visit and lasts until evidence of disease relapse, study termination, patient loss to follow-up, or withdrawal of the informed consent by the patient. In any case the follow-up phase will end after one year from the completion of study treatment of the last patient

enrolled. Patients will be followed every 3 months up to 1 year. The following assessments will be performed at the Follow Up Visit(s) (every 3 months) ±14 days:

- Clinical assessment: performance status (ECOG or Karnofsky).
- Physical examination, weight and vital signs (body temperature, pulse and blood pressure).
- Tumour assessment: brain MRI.
- Blood sampling for secondary and translational endpoints.
- Adverse events assessments (limited to adverse events considered related to the study treatment, as specified in section 10.3).

After disease relapse only survival contacts every two months are required (until death or end of study).

5.9 Definitions

End of treatment:
The end of treatment is 30 days after the last study treatment administration.
End of study:
The end of study is the date of death of the patient or the date of closure of the study.
Evaluable for toxicity:
All patients will be evaluable for toxicity from the time of their first treatment with the study drug (first dose of DC vaccine).
Evaluable for Response:
All patients will be evaluable for PFS if they have performed all 4 induction DC vaccinations.

5.10 Discontinuation/ Withdrawal of Participants from Study Treatment

Each participant has the right to withdraw study at any time. In addition, the investigator may discontinue a participant from the study at any time if the investigator considers it necessary for any reason including:

- Disease progression which requires discontinuation of the study medication or results in inability to continue to comply with study procedures.
- Ineligibility (either arising during the study or retrospective having been overlooked at screening).
- General or specific changes in the patient's condition render the patient unacceptable for further treatment in the judgment of the investigator.
- Significant protocol deviation.
- Significant non-compliance with treatment regimen or study requirements.
- An adverse event which requires discontinuation of the study medication or results in inability to continue to comply with study procedures.
- Pregnancy.
- Consent withdrawn.
- Lost to follow up.

In addition, the Investigator has the right to discontinue a participant from the study at any time if

considered necessary to protect patients' rights and health.

If any patient withdraws consent to the study, no further study procedure may be performed and no further datum may be collected. Previously available data, however, may still be used and analyzed as appropriate (e.g. toxicity data). Patients can withdraw consent at any time without giving any reason; the Investigator, however, should ask and, if given, the reason will be recorded in the CRF.

The reason for withdrawal will be recorded in the CRF.

If the participant is withdrawn due to an adverse event, the investigator will arrange for follow-up visits or telephone calls until the adverse event has resolved or stabilised.

5.11 Source Data

Source documents are original documents, data, and records from which participants' CRF data are obtained. These include, but are not limited to, hospital records (from which medical history and previous and concurrent medication may be summarised into the CRF), clinical and office charts, laboratory and pharmacy records, diaries, microfiches, radiographs, and correspondence.

CRF entries will be considered source data if the CRF is the site of the original recording (e.g., there is no other written or electronic record of data). All documents will be stored safely in confidential conditions. On all study-specific documents, other than the signed consent, the participant will be referred to by the study participant number/code, not by name.

Data on pre-screening leukapheresis and standard radiochemotherapy (except to assessment date for leukapheresis and last date of treatment administration for Stupp regimen) will not be recorded on eCRFs. Screening assessments must be all recorded in eCRF; laboratory assessments during treatment period or follow-up (except to haemoglobin, haematocrit, platelet count, white blood cell count with absolute neutrophil count and absolute lymphocyte count) will not be recorded on eCRFs instead. However, all this data will be reported in the patient's hospital chart records.

- The laboratory notebooks will be the source documents for the parameters relating to biological investigations; in detail the following data related to the secondary biological endpoints will be appropriately recorded in the eCRF:
- The results of the IFN-γ ELISPOT assay at baseline and in concomitance with subsequent disease restagings per protocol.
- The plasma levels of pro-inflammatory/pro-angiogenic factors (IL-1beta, IL-6, IL-8, IL-10, IL-12p70, TNFalpha, VEGF and Fibronectin) at baseline and in concomitance with subsequent disease restagings per protocol.
- Comparison of tumor-associated antigens expression with PFS and OS.
- Relative proportion of T naïve, T central memory (TCM), T effector memory (TEM), T effector (TE),

MDSCs and Treg cells at the level of the peripheral blood before treatment, and in concomitance with subsequent disease restagings per protocol.
- T lymphocytes, MDSCs and Tregs at the level of the TME.

HLA results (optional), obtained by Genetic Lab. of Pievesestina, will be sent to Immuno-Gene Research & Therapy and archived in the laboratory notebooks. Physician or delegated staff will annotate this data in eCRF.

Direct access will be granted to authorised representatives from the sponsor/promoter, host institution and the regulatory authorities to permit trial-related monitoring, audits and regulatory inspections, including provision of direct access to source data and documents.

6. STUDY TREATMENT

The experimental treatment consists of an induction phase with 4 weekly doses of dendritic cell vaccine (10x106 cells) intradermally administered (weeks 1-4), followed by a maintenance phase consisting of 28 days cycles with vaccine administration (start week 7) and temozolomide (150-200mg/m2/day) assumed orally from day 1 to 5 q28 (start week5). The combined maintenance treatment will continue until disease progression, unacceptable toxicity or withdrawal of consent by the patient, or up to a maximum of 1 year of treatment. If the vaccine runs out, the experimental treatment continues with temozolomide alone. After disease progression or the end of maintenance phase, is foreseen a one-year follow-up phase for each subject.

6.1 Description of Study Treatment

The vaccine inoculation will start within 72 from registration date. Vaccine cycles will be divided on Induction phase (4 weekly doses, Wk1-4) and the Maintenance phase (28 days cycles: DC vaccine start week 7 and temozolomide (150-200mg/m2/day) from day 1 to 5, from Week 5, for a maximum of twelve cycles). The first cycle of TMZ will be 150 mg/m2/day and subsequent 200 mg/m2/day, as for clinical practice.

Table 1 Study treatment Description

Study Drug	Premedication; precautions	Dose	Route	Schedule
Dendritic Cell Vaccine	NA	$10x10^6$ cells	intradermal	Weeks: 1, 2, 3, 4, 7, 11, 15, ... and every 28 days
TMZ	NA	150-200 $mg/m^2/day$	orally	weeks: 5, 9, 13, … and every 28 days; from day 1 to 5 of every cycle

6.2 Supply of study treatment

6.2.1. Autologous DC vaccine

Autologous DC vaccine, produced under GMP condition by following specific SOPs of the Cell Factory, will be administered according to the following mode and schedule:

1. All vaccine doses will be from aliquots of at least 13x106 frozen dendritic cells in a with 90% autologous plasma and 10% DMSO.
2. starting from week 11, 3 additional doses will be administered weekly (Induction phase), then from week 18 doses will be every 21 days (Maintenance phase)

Each vaccine dose (10×106 total dendritic cells) is produced as described in paragraph 1.2 and it is provided by the Cell factory in 1-ml syringes. The content of each syringe is administered intradermally with 5 injections in sites close to inguinal or axillary lymph node stations that had not been site of previous surgical exeresis, preferentially alternating injection sites in consecutive vaccine administrations.

6.2.1.1. Product description

Each vaccine dose consists of 10×106 autologous dendritic cells loaded with autologous tumor homogenate, resuspended in sterile normal saline and dispensed in 2 insulin syringes. Each syringe is provided with a tag indicating the lot of the product to ensure traceability (according to GMP Vol4). As prescribed by Advanced Therapy Medicinal Products current regulations, all information concerning manufacturing of each vaccine dose, in particular those concerning traceability of the product, are recorded in Patient’s Batch Record.

6.2.1.2. Storage requirements

Each vaccine dose MUST be maintained for up to 2 hours at room temperature or 8 hours at 2-8°C.

6.2.1.3. Stability

Each vaccine dose MUST be administered to the patient within the shelf life which is 2 hours if stored at

room temperature and 8 hours if stored at 2-8°C.

6.2.1.4 Route of administration

The DC vaccine should be administered only intradermally by 5 injections in sites close to inguinal or axillary lymph node stations that had not been site of previous surgical exeresis. Whenever possible, vaccine administrations should be performed by alternating injection sites, avoiding to repeatedly administer consecutive vaccine doses at the same site. As an example, if first vaccine dose has been administered by 5 injections close to left inguinal LN stations and 5 injections close to right inguinal LN stations, the second vaccine should be given by 5 injections close to left axillary LN stations and 5 injections close to right axillary LN stations. The product is for intradermal injection only. It is not intended for administration by any other route.

6.2.2. Temozolomide

Institutions should follow their standard guidelines for temozolomide. Temozolomide is taken orally one a day for 5 days of each cycle during Maintenance Phase. For further details, refer to the SPC of temozolomide.

6.3 Compliance with Study Treatment

Patient must return Temozolomide drug boxes to the center to provide for the remaining capsules accountability.

6.4 Accountability of the Study Treatment

All dispensations of study medications will be documented.

Study pharmacist or personal delegated by PI will correctly complete and apply the study labels on TMZ drug boxes and to the “patient treatment preparation form” for the dendritic vaccine (a specific box doesn’t exist). The drug accountability forms are to be completed in a timely manner by the study staff and verified by the monitor. If feasible, electronic drug accountability form may be used (Log80).

6.5 Concomitant Medication

Throughout the study, Investigators may prescribe any concomitant medication or treatment deemed necessary to provide adequate supportive care. Corticosteroids dosing of > 20 mg prednisone daily are prohibited (except for those prescribed to treat immune related Adverse Events). If these are required, the participant will be withdrawn from study. Antiallergic prophylaxis for CT scans or MR contrast media is permitted.

Any medication other than the study medications prescribed and taken during the study will be recorded

in the eCRF until the end of treatment visit.

Any systemic corticosteroid, as well as any other drugs with known immunomodulatory activity, that is prescribed for treatment-emergent or disease-related medical conditions will be recorded on the CRF. The information on corticosteroids must include the name of the drug, dose, dosing frequency and any information relevant to the use of corticosteroids for treatment of treatment-related adverse events or emergent neurological complications. Investigators should add any clinical notes about the response to corticosteroid therapy as it relates to clinical or radiologic status changes.

7. DOSING DELAYS / DOSE MODIFICATIONS

Single vaccine doses might be anticipated or postponed by up to 3 weeks during treatment phase. If the delay is longer than 3 weeks, patient must be discontinued.

TMZ orally assumption might be anticipated or postponed by up to 3 weeks during maintenance phase. If the delay is longer than 3 weeks, patient must be discontinued.

No dose reduction will be performed in any case for DC vaccine as in our previously experience this treatment showed no clinically significant side effects.

Temozolomide dosing is based on adverse events (AEs) which occurred during the prior treatment cycle. If multiple AEs are seen, the dose administered should be based on the dose reduction required for the most severe grade of any single AE. On day 1 of each cycle (within the prior 72 hours), ANC > = 1.5 x 10/L, platelet count ≥100 x 10/L and all grade 3 or 4 non-hematologic AEs (except alopecia, nausea, and vomiting) must have resolved (to grade 1). If AEs persist, treatment should be delayed by 1 week for up to 3 consecutive weeks. If, after 3 weeks of delay, all AEs have still not resolved, then any further maintenance treatment with temozolomide should be stopped.

7.1 Monitoring and Toxicity Management

Each patient receiving first treatment with the study drug (first dose of DC vaccine) will be evaluable for safety. The safety parameters include all laboratory tests and hematological abnormalities, physical findings, and spontaneous reports of adverse events reported to the investigator by patients.

Each patient will be assessed periodically for the development of any toxicity as outlined in Section 5 Study Procedures. Toxicity will be assessed according to the NCI CTCAE v5.0.

8. BIOMARKER / CORRELATIVE / SPECIAL STUDIES (if applicable)

8.1 Materials and methods

<u>Paraffin embedded tissue sections</u> from the primary/metastatic tumor sample collected during screening phase are requested for the tumor microenvironment characterization and tumor antigens expression evaluation. The following procedures have to be followed:

Prepare paraffin embedding tissue slides:

- 1 hematoxylin-eosin section. The pathologist is required to mark the delimitation of the tumor areas on hematoxylin-eosin slide and to evaluate the percentage of tumor cells in these areas.
- a total of 5 white sections of 5µM placed on positive charge slides from the primary/metastatic tumor sample are required

<u>N° 5 sample tube (10 ml of blood) containing EDTA for PBMCs isolation</u> *(to be collected during pre-screening phase, screening, after induction phase and every 2 cycles of study treatment during maintenance phase, at EoT (if not performed at the previous treatment cycle) and during follow-up phase):*

1. Invert 2-3 times the tube to omogenize the sample.
2. Prepare the exact number of Leucosep® tubes required with 13 ml of LymphoSep® (lymphocyte separation medium).
3. Centrifuge at 250 g for 30 sec at 20°C to allow the LymphoSep® to pass under the filter of the Leucosep® tube.
4. Dilute the blood with a volume equal to 1 time (1:1 ratio) of RPMI medium (RPMI 1640 or HBSS) at room temperature and aliquot gently the diluted blood on the Leucosep® tubes previously prepared.
5. Centrifuge at 620 g for 10 minutes at 20°C (WITHOUT BRAKE).
6. Note: after recovering the samples set the centrifuge at + 4 °C.
7. Transfer each PBMC ring to a new 50 ml Falcon tube, minimizing the aspiration of Ficoll.
8. Dilute the cells collected with RPMI 1640 or HBSS up to a volume of 45 ml.
9. Centrifuge at 620 g for 10 min at 4°C.
10. Completely eliminate the supernatant, resuspend the cell pellets of that sample and combine them in a single 50 ml Falcon, making up to volume (45 ml) with RPMI or HBSS. Note: attention! after this first washing the pellet tends to flake easily, avoid decanting the supernatant to the end or if you prefer to vacuum.
11. Centrifuge at 200 g for 15 min at 4°C (to remove platelets).
12. Completely eliminate the supernatant and resuspend the cell pellet in the same volume of lysing solution for red blood cells (ACK Lysing Buffer).
13. Incubate 2-3 minutes at 4°C and make up to volume with RPMI (45 ml).
14. Centrifuge at 620 g for 10 min at 4°C.
15. Discard the supernatant and resuspend in 45 ml of RPMI.
16. Centrifuge at 620 g for 10 min at 4°C.
17. Discard the supernatant and resuspend in 45 ml of RPMI.
18. Count the cells and centrifuge the cell suspension at 620 g for 10 min at 4°C.
19. Prepare the cryopreservative solution (RPMI 60% + AB human serum 30% + 10% DMSO). Keep the solution cold (on ice). Prepare the fresh cryopreservative solution from time to time. Prepare 1mL of

cryopreserved solution for each vial you wish to freeze (e.g. 10 total vials = 10mL, * Prepare extra 1mL to avoid being lax with the last sample).

Aliquots of PBMCs to be stored for the immunological determinations:

- 10x10 ^ 6 cells_1 vial
- 5x10 ^ 6 cells_5 vials

the remaining cells_n. vials of 20 to 30x10 ^ 6 cells
(e.g. if the remaining cells are 22x10 ^ 6, make a single vial with that number of cells)

Prepare the vials to be frozen on ice and once the sample has aliquoted, immediately transfer the vials to the Freezing Box (CoolCell) at -80 C.
Within 72 hours, transfer the vials in a nitrogen vapor freezer until the required tests are performed.

N° 1 sample tube (4 ml of blood) containing EDTA for plasma isolation *(to be collected during pre-screening phase, screening, after induction phase and every 2 cycles of study treatment during maintenance phase, at EoT (if not performed at the previous treatment cycle) and during follow-up phase):*
invert 2-3 times the tube to omogenize the sample, centrifuge for 10 min at 1750 g at room temperature with brake, carefully collect the sovranatant in 15 ml falcon tubes and centrifuge for 10 min at 1750 g at room temperature with brake. Then transfer the sovranatant (plasma) in at least 2 aliquots of 500ul in cryovials. Label the vials as described follow and store them at -80°C until use.

N° 1 sample tube (4 ml of blood) containing EDTA (OPTIONAL): to be collected during screening phase; it will be shipped to Genetic Laboratory, Pievesestina (Cesena) for the HLA haplotype characterization.

8.3 Samples shipment

All biological samples will be anonimized: all patients enrolled will be assigned an identification code (subject ID) when registered on Openclinica platform, in order to maintain rigorous confidentiality standards.
The Coordinating Center will provide IRST Participating Center of the specific Operative Procedures.
The paraffin embedded tissue sections from the primary/metastatic tumor samples will be inquired at the pathological anatomy of Morgagni Hospital by the chief investigator of the clinical trial.
Blood samples for PBMCs isolation and plasma purification will be sent through internal procedures to IRST IRCCS Centro Risorse Biologiche (CRB), from Monday to Friday to the following address:

Dott.ssa Valentina Ancarani
c/o Centro Risorse Biologiche (CRB),
IRCCS Istituto Romagnolo per lo Studio dei Tumori “Dino Amadori” – IRST S.r.l.
Via Maroncelli 40,
47014 Meldola (FC).

e-mail: valentina.ancarani@irst.emr.it
Phone: +39 0543 739015

The blood samples for HLA haplotype characterization (OPTIONAL) will be sent to Genetic Laboratory of Pievesestina (Cesena), from Monday to Thursday to the following address:

Dott.ssa Laura Renzi
Lab. Unico Area Vasta Romagna
U.O. Genetica Medica,
E-mail: laura.renzi@auslromagna.it
Phone: +39 0547 394798, Fax: +39 0547 394801

The shipment of samples must be announced via e-mail laura.renzi@auslromagna.it including date of shipment.

8.4 Sample analysis, confidentiality and sample destruction

The biomolecular characterization of biological samples will be performed at the Bioscience Laboratory of IRCCS IRST and the parameters relating to biological investigations will be annotated on laboratory notebooks.
Genetic Laboratory of Pievesestina (Cesena) staff will send HLA haplotype results to IRCCS IRST Immuno-Gene Research & Therapy and they will be archived in the laboratory notebooks.
Genetic information obtained from patient samples will remain confidential.
Samples will be destroyed after 15 years from the end of the study and all associated data will be deleted from the study repository.

9. MEASUREMENT OF EFFECT

9.1 Efficacy Parameters

Progression-free survival
For the purpose of this study the relapse date is defined as the date when a clinically suspicious lesion is first identified by the Investigator. This means that, if further diagnostic activity (including follow-up assessment of a lesion) is required to confirm or rule out disease progression, the recurrence date should not be the date of final confirmation, but the date of first appearance of the lesion should be retrospectively assessed and considered as the relapse date.
Progression Free Survival (PFS) is calculated as the time from the date of leukapheresis to either disease progression, defined as above, or the date of death for any cause. Patients alive with no evidence of disease progression at the time of their last visit are censored at the time of the last examination.
Overall survival

Overall Survival (OS) is calculated as the time from the date of leukapheresis to the date of death for any cause. Patients still alive at the time of analysis are censored at the last time they are known to be alive.

9.2 Immunological endpoints assessment

Immunological efficacy will be assessed as a proportion of tumor-specific circulating immune effector cells determined by IFN-γ ELISPOT at baseline and in concomitance with subsequent disease restagings per protocol, after stimulation with peptide libraries fully covering the sequences of a panel of TAAs.

The definition of the prognostic or predictive role of the presence and/or development of these tumor-specific immune effector cells will be assessed. Moreover, the persistence of these anti-tumor specific effector cells after the completion of the vaccination program, both in relapsed and in disease-free patients, will be evaluated.

A selection TAAs already described as expressed in the investigated disease will be included and comprise: SOX2, SOX11, EZH2, TNC, BIRC5/SURVIVIN, IL13Ra2, HER2, IGF2BP3, MAGEA1, MAGEA3, GP100. Enhancement of at least 10% of antigen-specific spot-forming cells (SFC) subtracting spots obtained with unstimulated PBMC, for at least one antigen after treatment compared with baseline sample, will be considered as positive.

The plasma levels of pro-inflammatory/pro-angiogenics cytokines (IL-1beta, IL-6, IL-8, IL-10, IL-12, TNFalpha, VEGF and Fibronectin) will be evaluated in longitudinal plasma samples by cytometric bead array. The results will be analyzed to evaluate whether specific marker expression profiles, at the baseline and/or changes induced by vaccinations are able to predict the clinical outcome.

Additionally, functional phenotyping of circulating immune effectors/regulators will be conducted on PBMC samples taken before treatment, after the second vaccination and at the end of treatment and will be evaluated by multi-parametric flow cytometry as described in section 2.5. Data obtained will be analysed to evaluate changes in the frequency of these immune subsets upon DC vaccination. A correlation with clinical outcome will also be performed to verify whether specific circulating immune cell subtypes are predictive of efficacy.

IHC on FFPE tumour samples will be carried out to evaluate the expression of TAAs (such as SOX2, SOX11, EZH2, etc) and to identify main immune cell subsets, including T lymphocytes MDSCs and Tregs, within the TME. The following markers will be assessed: CD3, CD4, CD8, CD45RO, FOXP3, Granzyme B, TIA-1, CD11b, CD11c, CD14, CD163, HLA-class I, HLA-Class II. Then, these data will be analysed by parametric or non-parametric statistical tests as appropriate to evaluate their predictive role.

9.3 DTH skin test

The immunologic efficacy, i.e. the ability of the different treatments to efficiently induce antitumor immune responses, will be measured at the bedside by DTH skin test against tumor homogenate and KLH.

DTH testing will be performed in all patients on day 0 (pre-treatment DTH, which showed no reactivity in the large majority of patients so far evaluated in our previous studies), before the first cycle of Maintenance phase (post-treatment DTH) and every 2 cycles of vaccine. The diameter of induration/erythema observed after 24-48 hours is recorded according to the following scale: 0-5 mm grade 1, 6-10 mm grade 2, 11-20 mm grade 3, > 20 mm grade 4.

9.4 Method and Timing

Response and progression in this study will be evaluated using the international criteria proposed by the Response Evaluation Criteria in Brain Tumors Committee (Response Assessment in Neuro-Oncology guidelines, RANO) (see appendix D) associated to Perfusion MRI by DSC (dynamic susceptibility contrast).
MRI imaging acquisition protocol:
MRI will be performed with the INGENIA Philips 3T.
The protocol includes the following sequences:
FFE T1w 3D (thk 1 mm), T2 FLAIR 3D brain view (thk 1.12 mm), DWI EPI SPIR (thk 3 mm; B0-B1000); Coronal TSE T2w (thk 3 mm), FFE T2w (thk 4 mm), Perfusion MRI by DSC (dynamic susceptibility contrast) and FFE T1w 3D (thk 1mm) post paramagnetic contrast medium (Gadoteric Acid). A contrast volume of 0.2 mL / kg + 3 mL at 3.5 mL / s is injected for the DSC perfusion sequence.
DSC post processing:
The T2 * MRI signal intensity obtained during the dynamic administration of the contrast medium is converted into an intensity-time curve. The time-dependent signal intensity data, by means of a deconvolution analysis, are used to generate a colorimetric map of the cerebral blood volume (CBV). The variations of this parameter have a linear correlation with the presence of neoangiogenesis and the glial tumors grading[24-27]
DSC-MRI Imaging Analysis:
The colorimetric map of the rCBV will be processed and superimposed on the T1 sequence with contrast medium and the FLAIR sequence. For the quantitative analysis of the lesion, 5 freehand ROIs of 20 mm2 are drawn which express the value of the CBV ratio of the lesion and CBV of the white matter in the contralateral cerebral hemisphere. Hemorrhagic areas, vessels and areas characterized by suscepyibility artifacts are excluded. An rCBV value> 2.5, according to previous studies, is considered pathological.
The radiologist reporting the DSC MRI will be blinded to the results of RANO criteria, current reference criteria for the evaluation of glioblastoma (GBM).
The perfusion analysis will be performed in blind by two neuroradiologists with different experience:
a neuroradiologist with experience in GBM-MRI studies <5 years and a neuroradiologist with GBM-MRI studies experience > 20 years.
We want to try to distinguish between real progression of GBM from radionecrosis and pseudo progression by evaluating the 5 ROIs of the perfusion study and correlate them with RANO imaging criteria. In particular we want to demonstrate that the presence in at least 3 ROIs of rCBV values> 2.5 identifies real disease progression if in accordance with RANO imaging criteria.
Techniques used to measure disease should be the most accurate, reliable and reproducible methods that are routinely used.
For the purposes of this study, patients should be re-evaluated for response at the end of induction phase and every 2 cycles during maintenance phase.

10. SAFETY REPORTING

Analyses will be performed for all patients having received at least one dose of study treatment. The

National Cancer Institute's Common Terminology Criteria for Adverse Events (CTCAE) Version 5.0 should be used to assess and grade AE severity, including laboratory abnormalities judged to be clinically significant. If the experience is not covered in the modified criteria, the guidelines shown in the table below should be used to grade severity. It should be pointed out that the term "severe" is a measure of intensity and that a severe AE is not necessarily serious.

AE Severity Grading

Severity (Toxicity Grade)	Description
**Mild (1)**	Transient or mild discomfort; no limitation in activity; no medical intervention or therapy required. The subject may be aware of the sign or symptom but tolerates it reasonably well.
**Moderate (2)**	Mild to moderate limitation in activity, no or minimal medical intervention/therapy required.
**Severe (3)**	Marked limitation in activity, medical intervention/therapy required, hospitalizations possible.
**Life-threatening (4)**	The subject is at risk of death due to the adverse experience as it occurred. This does not refer to an experience that hypothetically might have caused death if it were more severe.

10.1 Definitions

Adverse Event (AE): Any untoward medical occurrence in a patient or clinical investigation participants administered a medicinal product, which does not necessarily have to have a causal relationship with this treatment (the study medication).

An AE can therefore be any unfavourable and unintended sign (including an abnormal laboratory finding), symptom or disease temporally associated with the use of the study medication, whether or not considered related to the study medication.

Adverse Reaction (AR): All untoward and unintended responses to a medicinal product related to any dose.

The phrase "responses to a medicinal products" means that a causal relationship between a study medication and an AE is at least a reasonable possibility, i.e., the relationship cannot be ruled out.

All cases judged by either the reporting medically qualified professional or the sponsor as having a reasonable suspected causal relationship to the study medication qualify as adverse reactions.

Severe Adverse Events: To ensure no confusion or misunderstanding of the difference between the terms "serious" and "severe", which are not synonymous, the following note of clarification is provided:

The term "severe" is often used to describe the intensity (severity) of a specific event (as in mild, moderate, or severe myocardial infarction); the event itself, however, may be of relatively minor medical significance (such as severe headache). This is not the same as "serious," which is based on patient/event outcome or action criteria usually associated with events that pose a threat to a participant's life or functioning. Seriousness (not severity) serves as a guide for defining regulatory reporting obligations.

Serious Adverse Event (SAE) or Serious Adverse Reaction: A serious adverse event or reaction is any untoward medical occurrence that at any dose:

- Results in death,

- Is life-threatening*,
- Requires inpatient hospitalisation or prolongation of existing hospitalisation**,
- Results in persistent or significant disability/incapacity, or
- Is a congenital anomaly/birth defect.
- Or is otherwise considered medically significant by the Investigator***

Comments:

The term severe is often used to describe the intensity (severity) of a specific event. This is not the same as serious, which is based on patients/event outcome or action criteria.

* Life threatening in the definition of an SAE refers to an event in which the patient was at risk of death at the time of the event; it does not refer to an event that hypothetically might have caused death if it were more severe.

**Hospitalisation is defined as an unplanned, formal inpatient admission, even if the hospitalisation is a precautionary measure for continued observation. Thus, hospitalisation for protocol treatment (e.g. line insertion), elective procedures (unless brought forward because of worsening symptoms) or for social reasons (e.g. respite care) are not regarded as an SAE.

*** Medical judgment should be exercised in deciding whether an AE is serious in other situations. Important AEs that are not immediately life threatening or do not result in death or hospitalisation but may jeopardise the subject or may require intervention to prevent one of the other outcomes listed in the definition above, should be considered serious.

Suspected Unexpected Serious Adverse Reactions (SUSAR): A serious adverse reaction, the nature or severity of which is not consistent with the applicable product information (e.g., Investigator's Brochure for an unapproved investigational product or package insert/summary of product characteristics for an approved product).

Expected Serious Adverse Events/Reactions:

Based on extensive clinical experience, no serious adverse events/reaction is reasonably expected during the course of the study or expected from the study medications.

Adverse Event Reporting Period

The study period during which adverse events must be reported is defined as the period from the first study treatment administration to the end of the study treatment follow-up. For this study, the study treatment follow-up is defined as 30 days following the last administration of study treatment.

Preexisting Condition

A preexisting condition is one that is present at the start of the study. A preexisting condition should be recorded as an adverse event if the frequency, intensity, or the character of the condition worsens during the study period.

General Physical Examination Findings

At screening, any clinically significant abnormality should be recorded as a preexisting condition. At the end of the study, any new clinically significant findings/abnormalities that meet the definition of an adverse event must also be recorded and documented as an adverse event.

Post-study Adverse Event

All unresolved adverse events should be followed by the investigator until the events are resolved, the

subject is lost to follow-up, or the adverse event is otherwise explained. At the last scheduled visit, the investigator should instruct each subject to report any subsequent event(s) that the subject, or the subject's personal physician, believes might reasonably be related to participation in this study. The investigator should notify the study sponsor/promoter of any death or adverse event occurring at any time after a subject has discontinued or terminated study participation that may reasonably be related to this study. The sponsor/promoter should also be notified if the investigator should become aware of the development of cancer or of a congenital anomaly in a subsequently conceived offspring of a subject that has participated in this study.

Abnormal Laboratory Values

A clinical laboratory abnormality should be documented as an adverse event if <u>any one of the following</u> conditions is met:

- The laboratory abnormality is not otherwise refuted by a repeat test to confirm the abnormality
- The abnormality suggests a disease and/or organ toxicity
- The abnormality is of a degree that requires active management; e.g. change of dose, discontinuation of the drug, more frequent follow-up assessments, further diagnostic investigation, etc.

Hospitalization, Prolonged Hospitalization or Surgery

Any adverse event that results in hospitalization or prolonged hospitalization should be documented and reported as a serious adverse event unless specifically instructed otherwise in this protocol. Any condition responsible for surgery should be documented as an adverse event if the condition meets the criteria for and adverse event.

Neither the condition, hospitalization, prolonged hospitalization, nor surgery are reported as an adverse event in the following circumstances:

- Hospitalization or prolonged hospitalization for diagnostic or elective surgical procedures for a preexisting condition. Surgery should ***not*** be reported as an outcome of an adverse event if the purpose of the surgery was elective or diagnostic and the outcome was uneventful.
- Hospitalization or prolonged hospitalization required to allow efficacy measurement for the study.
- Hospitalization or prolonged hospitalization for therapy of the target disease of the study, unless it is a worsening or increase in frequency of hospital admissions as judged by the clinical investigator

10.2 Reporting Procedures for All Adverse Events

All AEs occurring during the study observed by the investigator or reported by the participant, whether or not attributed to study medication, will be recorded on the CRF.

The following information will be recorded: description, date of onset and end date, severity (according to CTCAE version 5.0), assessment of relatedness to study medication, other suspect drug or device and action taken. Follow-up information should be provided as necessary.

AEs considered related to the study medication as judged by a medically qualified investigator or the sponsor/promoter will be followed until resolution or the event is considered stable. All related AEs that

result in a participant's withdrawal from the study or are present at the end of the study, should be followed up until a satisfactory resolution occurs.

Serious adverse events that are still ongoing at the end of the study period must be followed up to determine the final outcome. Any serious adverse event that occurs after the study period and is considered to be possibly related to the study treatment or study participation should be recorded and reported immediately.

It will be left to the investigator's clinical judgment whether or not an AE is of sufficient severity to require the participant's removal from treatment. A participant may also voluntarily withdraw from treatment due to what he or she perceives as an intolerable AE. If either of these occurs, the participant must undergo an end of study assessment and be given appropriate care under medical supervision until symptoms cease or the condition becomes stable.

The relationship of AEs to the study medication will be assessed by a medically qualified investigator.

AE Relationship to Study Drug

Relationship to Drug	Comment
**Definitely**	Previously known toxicity of agent; or an event that follows a reasonable temporal sequence from administration of the drug; that follows a known or expected response pattern to the suspected drug; that is confirmed by stopping or reducing the dosage of the drug; and that is not explained by any other reasonable hypothesis.
**Probably**	An event that follows a reasonable temporal sequence from administration of the drug; that follows a known or expected response pattern to the suspected drug; that is confirmed by stopping or reducing the dosage of the drug; and that is unlikely to be explained by the known characteristics of the subject's clinical state or by other interventions.
**Possibly**	An event that follows a reasonable temporal sequence from administration of the drug; that follows a known or expected response pattern to that suspected drug; but that could readily have been produced by a number of other factors.
**Unrelated**	An event that can be determined with certainty to have no relationship to the study drug.

Any pregnancy occurring during the clinical study and the outcome of the pregnancy, should be recorded and followed up for congenital abnormality or birth defect.

10.3 Reporting Procedures for Serious Adverse Events

The Investigator is responsible for reporting all Serious Adverse Events (SAE), related or not to the study treatment, occurring from main informed consent signature and within 30 days of the last protocol treatment, to the "Safety Desk". Any late Serious Adverse Drug Reaction (SADR), occurring after this 30-day period, should follow the same reporting procedure.

If a SAE occurs, the following action must be taken by the investigator:

Fill in the SAE form and send by fax within 24 hours of the initial observation of the event, to the sponsor/promoter:

IRST Safety Desk

FAX 0543 739288

e-mail: fv.ct@irst.emr.it

- Attach a report of the event and a copy of all examinations that were carried out, including the dates on which these examinations were performed. For laboratory tests, normal laboratory ranges must also be included.
- All forms must be dated and signed by the responsible investigator or one of his/her authorized staff members.
- Additional information received for a case (follow-up or corrections to the original case) need to be detailed on a new SAE form and faxed to IRST.
- IRST Safety Desk will perform an initial check of the information and ensure that it is reviewed by the responsible safety physician.
- The IRST safety desk will send the SAE report to national authorities, Ethical Committees and investigators as appropriate, according to local regulations.
- IRST will report all SUSARs to the Competent Authorities and the Ethical Committees concerned. Fatal or life-threatening SUSARs must be reported within 7 days and all other SUSARs within 15 days. IRST will also inform all investigators concerned of relevant information about SUSARs that could adversely affect the safety of participants.
- In addition to the expedited reporting above, the IRST Safety Desk shall submit once a year throughout the clinical trial or on request a safety report to the Competent Authority and Ethical Committees.

11. STATISTICAL CONSIDERATIONS

Prior to the analysis of the final study data, a detailed Statistical Analysis Plan (SAP) will be written describing all analyses that will be performed. The SAP will contain any modifications to the analysis plan described below.

11.1 Study Design/Endpoints

This is a single-arm, monocentric, phase II study, to evaluate the safety and the progression-free survival related to the combined treatment of dendritic cell vaccine loaded with autologous tumor homogenate and temozolomide in patients operated for glioblastoma and then treated with standard radiochemotherapy (according to Stupp regimen).

To avoid unacceptable toxicity, a formal safety analysis will be conducted after the enrollment of 9 evaluable for toxicity patients included in the first step of the study. These patients have been observed for at least 30 days after the end of induction phase. If three or more patients have experienced grade 3 or higher adverse events related to the study treatment, enrollment will be definitively stopped;

differently, the other patients to complete study recruitment, will be enrolled.
Primary endpoint of the study will be PFS, calculated time from the date leukapheresis to the date of first progression or the date of death from any cause or the date of the last restaging in non-progressed patients. For the evaluation of primary objective, the proportion of patients without progression of disease at three months after the date of leukapheresis will be calculated.
Safety of study treatment will be considered as co-primary endpoint: the adverse events recorded during the observation period (i.e. from the day of the first DC vaccine administration up to 30 days after the last dose of study treatment), will be reported and graded according to NCI CTCAE 5.0, their severity and causal relationship with the investigational products will be analyzed by means of descriptive statistics.
The OS is a secondary objective and will be measured from the date of leukapheresis until the date of death from any cause or the last date on which it was known that the patient was alive.
Other immunological objectives are defined and descriptive measures will be calculated to analyse them.

11.2 Sample Size, Accrual Rate and Study Duration

Simon's two-stage design (Simon, 1989) will be used to evaluate the primary objective. The null hypothesis that the proportion of patients without progression of disease at 3 months from the leukapheresis date is 70% will be tested against a one-sided alternative. In the first stage, 9 evaluable patients for response will be accrued. If there are 6 or fewer patients without progression of disease at 3 months from the date of leukapheresis in these 9 patients, the study will be stopped. Otherwise, 19 additional evaluable patients will be accrued for a total of 28. The null hypothesis will be rejected if 22 or more patients without progression of disease at three months are observed in 28 patients. This design yields a type I error rate of 10% and power of 80% when the true proportion of patients is 87%.

- Estimated duration for the main protocol: 55 months
- Enrolment: 30 months
- Treatment period: 13 months
- Follow up: 12 months for each patient

11.3 Stratification Factors

N.A.

11.4 Analysis of Primary Endpoints

Time to events (PFS and OS) will be calculated with the Kaplan-Meier method and the analysis was performed on the eligible population. The proportion of patients experiencing treatment-related grade ≥ 3 AEs during the treatment will be inferred by means of the two-sided Clopper-Pearson, or a more appropriate one, 95% confidence interval.

11.5 Analysis of Secondary Endpoints

OS analysis calculation is described in 11.4 paragraph. Evaluation of DTH at different timepoints will be evaluated considering a repeated measures scenario. Descriptive statistics will be used to assess the extent of the secondary endpoints: frequency tables will be performed for all categorical variables. Continuous variables will be presented using mean and standard deviation or median and range.

11.6 Interim Analysis and Criteria for termination of the trial

A planned interim analysis will be done after the recruitment of the first 9 evaluable patients for toxicity (at least 30 days after the induction phase of the 9th evaluable patient enrolled). Stopping rules are defined as follow: if three or more patients had an experience of treatment-related grade ≥ 3 AEs the study will be stopped due to lack of safety, while if there are 6 or fewer responses after the recruitment of the first 9 evaluable patients for response (at least after three months from the leukapheresis date of the 9th evaluable patient enrolled) will be stopped due to lack of efficacy.

11.7 Reporting and Exclusions

Evaluation of toxicity.
The patients will be evaluable for toxicity if they perfomed at least one DC vaccination during the induction phase.
Evaluation of response.
The patients will be evaluable for response if they have performed all 4 induction DC vaccinations.

11.8 Procedure(s) to account for missing or spurious data

To limit the presence of missing data, the number of follow-up visits are minimized, collecting only the essential information at each visit, and developing the user-friendly case-report forms.

Furthermore, before the beginning of the clinical research, a detailed documentation of the study which includes the methods to screen the participants, protocol to train the investigators, implementation of the treatment, and procedure to collect, enter, and edit data, will be implemented.

Before the start of the participant enrollment, a training should be conducted to instruct all personnel related to the study on all aspects of the study, such as the participant enrollment, collection and entry of data, and implementation of the treatment.

The data collection will be monitored and reported in as close to real-time as possible during the course of the study.

Finally, if a patient decides to withdraw from the follow-up, the reasons for the withdrawal should be recorded for the subsequent analysis in the interpretation of the results.

11.9 Other statistical considerations.

Any deviation from the original statistical plan will be discussed in the working study team and, if necessary, the protocol will be modified.

12. ETHICAL ASPECTS

12.1 Declaration of Helsinki

The Investigator will ensure that this study is conducted in full conformity with the current revision of the Declaration of Helsinki (last amended 64th WMA General Assembly, Fortaleza, Brazil, October 2013).

12.2 ICH Guidelines for Good Clinical Practice

The Investigator will ensure that this study is conducted in full conformity with relevant regulations and with the ICH Guidelines for Good Clinical Practice (CPMP/ICH/135/95) July 1996, Regulation (EU) n. 536/2014 of the European Parliament and other relevant local legislation.

12.3 Independent Ethical Committee (IEC)

The protocol, informed consent and any accompanying material provided to the patient will be submitted by the investigator to an Independent Ethical Committee for review. Approval from the committee must be obtained before starting the study. Any modifications made to the protocol, informed consent or material provided to the patient after receipt of the Ethics Committee approval must also be submitted by the investigator to the Committee in accordance with local procedures and regulatory requirements. The IEC approval report must contain details of the trial (title, protocol number and version), documents evaluated (protocol, informed consent material) and the date of the approval.

12.4 Informed Consent

It is the responsibility of the Investigator to obtain written informed consent from each subject prior to entering the trial or, where relevant, prior to evaluating the subject's suitability for the study.The informed consent document used by the Investigator for obtaining the subject's informed consent must be reviewed and approved by the IEC.
All patients will be informed of the aims of the study, the possible adverse events, the procedures and possible hazards to which he will be exposed, and the mechanism of treatment allocation. In obtaining and documenting informed consent, the investigator should comply with the applicable regulatory requirement(s), and should adhere to Good Clinical Practice (GCP) and to ethical principles that have their origin in the Declaration of Helsinki.
It will be emphasized that the participation is voluntary and that the patient is allowed to refuse further participation in the protocol whenever he wants. This will not prejudice the patient’s subsequent care.

Documented informed consent must be obtained for all patients included in the study before they are registered for the study. This must be done in accordance with the national and local regulatory requirements.
The written informed consent form should be signed and personally dated by the patient or by the patient's legally acceptable representative. The clinical records for each patient shall document the informed consent process and that written informed consent was obtained prior to participation in the study. A copy of the signed informed Consent Form must be provided to the patient or the patient's legally authorized representative. The original copy of the patient's signed written consent will be kept by the center in the proper section of the Investigator Site File and must be available for verification by study monitors at any time.

12.5 Patient data protection

The Informed Consent Form will incorporate (or, in some cases, be accompanied by a separate document) wording that complies with relevant data protection and privacy legislation. In agreement with this wording, patients will authorize the collection, use and disclosure of their study data by the Investigator and by those persons who need that information for the purposes of the study.

The Informed consent Form will explain that the study data will be stored in a computer data base, maintaining confidentiality in accordance with national data legislation.
The Informed Consent Form will also explain that for data verification purposes, authorized representatives of Sponsor/Promoter, a regulatory authority, an Ethics Committee may require direct access to parts of the hospital or practice records relevant to the study, including patients' medical history.

13. DATA COLLECTION

The Investigator will prepare and maintain adequate and accurate source documents designed to record all observations and other pertinent data for each subject treated with the study drug.
Study personnel at each site will enter data from source documents corresponding to a subject's visit into the protocol-specific electronic Case Report Form (eCRF) OR paper CRF when the information corresponding to that visit is available. Subjects will not be identified by name in the study database or on any study documents to be collected by the Sponsor/Promoter (or designee), but will be identified by a site number, subject number.

Electronic Case Report Forms

eCRFs are to be completed through use of a Sponsor-designated EDC system. Sites will receive training and have access to a manual for appropriate eCRF completion. eCRFs will be submitted electronically to the Sponsor/Promoter and should be handled in accordance with instructions from the Sponsor/Promoter. All eCRFs should be completed by designated, trained site staff. eCRFs should be reviewed and electronically signed and dated by the investigator or a designee.

If a correction is required for an eCRF, the time and date stamps track the person entering or updating eCRF data and creates an electronic audit trail.
At the end of the study, the investigator will receive patient data for his or her site in a readable format on a compact disc that must be kept with the study records. Acknowledgement of receipt of the compact disc is required.

14. STUDY MONITORING

The Investigator(s) agree to(s) to perform the study in accordance with ICH Good Clinical Practice.
The Investigator is required to ensure his compliance to the procedures required by the protocol with respect to the investigational drug schedule and visit schedule. The Investigator agrees to provide all information requested in the Case Report Form in an accurate and legible manner according to the instructions provided.
The Investigator has responsibilities to the Health Authorities to take all reasonable steps to ensure the proper conduct of the study as regards ethics, protocol adherence, integrity and validity of the data recorded on the case report forms.

14.1 Site Set-up and Initiation

All participating Investigators will be asked to sign the necessary agreements and supply a current CV to the coordinating center or Sponsor/Promoter.
All members of the site research team will also be required to sign the "*Site Signature and Delegation Log*".
Prior to commencing recruitment all sites will undergo a process of initiation. Key members of the site research team will be required to attend either a meeting or a teleconference covering aspects of the trial design, protocol procedures, Adverse Event reporting, collection and reporting of data and record keeping.
Sites will be provided with an Investigator Site File containing essential documentation, instructions, and other documentation required for the conduct of the trial. The coordinating centre or Sponsor/Promoter must be informed immediately of any change in the site research team.

14.2 On-site Monitoring

If a monitoring visit is required the coordinating center, or Sponsor/Promoter will contact the site to arrange a date for the proposed visit and will provide the site with written confirmation. Investigators will allow the trial staff access to source documents as requested.
The main duty of the Trial Monitor is to help the Investigator and the Study Coordinators to maintain a high level of ethical, scientific, technical and regulatory quality in all aspects of the study.
The first monitoring visit will take place within 30 days of enrollment of the first patient.
Subsequent periodic monitoring visits will be performed on a 4-weekly basis, adjustable according to patient recruitment and in accordance with study staff.

During each monitoring visit, the following points will be checked: subject informed consent, subject recruitment and follow-up, study drug allocation, subject compliance to the study treatment, study treatment accountability, Adverse Event documentation and reporting.
According to the guidelines on ICH Good Clinical Practice, the trial monitor will check the case report form entries against the source documents. These personnel, bound by professional secrecy, will not disclose any personal identity or personal medical information.

14.3 Central Monitoring

Trials staff will be in regular contact with the site research team to check on progress and address any queries that they may have. Trials staff will check data received for compliance with the protocol, data consistency, missing data and timing. Sites will be sent requests missing data or clarification of inconsistencies or discrepancies. For eCRF trials these requests may be generated by automated data validation checks.
Adverse events of grade ≥ 3 related to the experimental treatment must be promptly recorded in eCRF and reported to the CdC (within 3 days of the initial observation of the event).
Close central monitoring will be performed by the CdC study coordinator upon registration and afterwards every 4 weeks after the first DC vaccine administration on all safety data.
All safety data should be monitored on site before safety analysis, which will be conducted after the first 9 patients evaluable for toxicity have been observed for at least 30 days after the induction phase of the 9th subject. Stopping rules are defined as follow: if three or more patients had an experience of treatment-related grade ≥ 3 AEs the study will be stopped due to lack of safety, while if there are 6 or fewer responses after the recruitment of the first 9 patients evaluable for response (at least after three months from the leukapheresis date of the 9th evaluable patient enrolled), the trial will be stopped due to lack of efficacy. At these timepoints the patient enrollment will be interrupted: enrollment of 9 patients evaluable for toxicity and 9 patients evaluable for response.

14.4 Audit and Inspection

The Investigator will permit trial-related monitoring, audits, ethical review, and regulatory inspection(s) at their site, providing direct access to source data/documents.
Sites are also requested to notify the coordinating center or Sponsor/Promoter of any CA inspections.

15. ADMINISTRATIVE REGULATIONS

The CC is responsible for drawing up the final version of the protocol, implementing the CRFs and updating the electronic database, defining general organizational procedures and organizing periodic meetings and newsletters. The CC will also undertake the following: support for the preparation of all documents needed for EC submission of the study protocol for each participating center, training of staff assigned to data collection, definition of monitoring procedures and monitor training.

15.1 Curriculum vitae

An updated copy of the curriculum vitae of each Principal Investigator, duly signed and dated, will be provided to the study monitor prior to the beginning of the study.

15.2 Secrecy agreement

All goods, materials, information (oral or written) and unpublished documentation provided to the Investigators, including this protocol and the case report forms, shall be considered confidential and may not given or disclosed to third parties.

15.3 Availability and Retention of Investigational Records

The Investigator must make study data accessible to the monitor, other authorized representatives of the Sponsor/Promoter (or designee), IEC, and Regulatory Agency inspectors upon request. A file for each subject must be maintained that includes the signed Informed Consent and copies of all source documentation related to that subject. The Investigator must ensure the reliability and availability of source documents from which the information on the CRF was derived.

All study documents (patient files, signed informed consent forms, copies of CRFs, Study File Notebook, etc.) must be kept secured for a period of 15 years after completion or discontinuation of the study or for the length of time required by relevant national or local health authorities, whichever is longer. There may be other circumstances for which the Sponsor/Promoter is required to maintain study records and, therefore, the Sponsor/Promoter should be contacted prior to removing study records for any reason.

15.4 Insurance

A clinical trial insurance has been arranged, according to the Italian law (DM of 14th of July 2009) for this specific trial. The clinical trial insurance is only valid if treatment is given in a center authorized by IRST IRCCS and which has obtained Ethical Committee approval. All daily clinical practice procedures refer to the business insurance of the participating center where the patient is treated.

EU n. 536/2014 states:

Member States shall ensure that systems for compensation for any damage suffered by a subject resulting from participation in a clinical trial conducted on their territory are in place in the form of insurance, a guarantee, or a similar arrangement that is equivalent as regards its purpose and which is appropriate to the nature and the extent of the risk.

The sponsor and the investigator shall make use of the system referred to above in the form appropriate for the Member State concerned where the clinical trial is conducted.

Member States shall not require any additional use of the system referred to in paragraph 1 from the sponsor for low-intervention clinical trials, if any possible damage that could be suffered by a subject resulting from the use of the investigational medicinal product in accordance with the protocol of that specific clinical trial on the territory of that Member State is covered by the applicable compensation system already in place.

16. OWNERSHIP OF DATA AND USE OF THE STUDY RESULTS

The full ownership of the data generated in this study is retained by IRST and by all the investigators actively recruiting patients.
Data deriving from this clinical trial are not intended for drug registration or for patent applications, but only for scientific and educational purposes, which include presentation at scientific meetings, congresses and symposia and/or publication in scientific journals.

17. PUBLICATION POLICY

Publications regarding the main study end-points will be prepared by the Chief Investigator. Authorship will be proportional to the accrual of each center. All the members of the steering committee and components of the CC will be included in the authors list and all the investigators recruiting will be mentioned as contributors. Other area-specific publications will be prepared by the coordinators of the single treatment modalities to increase the visibility of the study and investigators. However, the publication of secondary endpoints is discouraged before publication of the main endpoint and should be anyway discussed with the study and writing committee coordinators.

18. PROTOCOL AMENDMENTS

It is specified that the appendices, attached to this protocol and referred to in the main text of this protocol, form an integral part of the protocol.
No changes or amendments to this protocol may be made by the Investigators after the protocol has been agreed to and signed by both parties .Any change agreed upon will be recorded in writing, the written amendment will be signed by the Chief Investigator and by the Principal Investigator and the signed amendment will be appended to this protocol.
Approval / advice of amendments by Ethical Committees or similar body is required prior to their implementation, unless there are overriding safety reasons.
If the change or deviation increases risk to the study population, or adversely affects the validity of the clinical investigation or the subject's rights, full approval / advice must be obtained prior to implementation. For changes that do not involve increased risk or affect the validity of the investigation or the subject's rights, approval / advice may be obtained by expedited review, where applicable.
In some instances, an amendment may require a change to a consent form. The Investigator must receive approval / advice of the revised consent form prior to implementation of the change.

19. REFERENCES

1. Ostrom QT, Bauchet L, Davis FG, Deltour I, Fisher JL, Langer CE, Pekmezci M, Schwartzbaum JA, Turner MC, Walsh KM, Wrensch MR, Barnholtz-Sloan JS.The epidemiology of glioma in adults: a "state of the science" review. Neuro Oncol. 2014 Jul;16(7):896-913
2. Stupp R, Hegi ME, Mason WP, van den Bent MJ, et al,; European Organisation for Research and Treatment of Cancer Brain Tumour and Radiation Oncology Groups; National Cancer Institute of Canada Clinical Trials Group. Effects of radiotherapy with concomitant and adjuvant temozolomide versus radiotherapy alone on survival in glioblastoma in a randomised phase III study: 5-year analysis of the EORTC-NCIC trial. Lancet Oncol. 2009 May;10(5):459-66. doi: 10.1016/S1470-2045(09)70025-7.
3. Stupp R, Taillibert S, Kanner AA, et al. Maintenance Therapy With Tumor-Treating Fields Plus Temozolomide vs Temozolomide Alone for Glioblastoma: A Randomized Clinical Trial JAMA. 2015 Dec 15;314(23):2535-43
4. De Felice F, Musio D, Cassese R, Gravina GL, Tombolini V.New Approaches in Glioblastoma Multiforme: The Potential Role of Immune- check Point Inhibitors.Curr Cancer Drug Targets. 2017;17(3):282-289.
5. Kurz SC1, Wen PY2. Quo Vadis-Do Immunotherapies Have a Role in Glioblastoma? Curr Treat Options Neurol. 2018 Apr 18;20(5):14.
6. Palucka K, Banchereau J.Cancer immunotherapy via dendritic cells.
Nat Rev Cancer. 2012 Mar 22;12(4):265-77.
7. Palucka K, Banchereau J Dendritic-cell-based therapeutic cancer vaccines. Immunity. 2013 Jul 25;39(1):38-48
8. Huber A, Dammeijer F, Aerts JGJV, Vroman H.Current State of Dendritic Cell-Based Immunotherapy: Opportunities for *in vitro* Antigen Loading of Different DC Subsets? Front Immunol. 2018 Dec 3;9:2804.
9. Rodríguez Pérez Á, Campillo-Davo D, Van Tendeloo VFI, Benítez-Ribas D. Cellular immunotherapy: a clinical state-of-the-art of a new paradigm for cancer treatment. Clin Transl Oncol. 2020 Apr 7. doi: 10.1007/s12094-020-02344-4
10. Polyzoidis S, Tuazon J, Brazil L, Beaney R, Al-Sarraj ST, Doey L, Logan J, Hurwitz V, Jarosz J, Bhangoo R, Gullan R, Mijovic A, Richardson M, Farzaneh F, Ashkan K. Active dendritic cell immunotherapy for glioblastoma: Current status and challenges. Br J Neurosurg. 2015 Apr;29(2):197-205
11. Liau LM, Ashkan K, Tran DD, Campian JL et al First results on survival from a large Phase 3 clinical trial of an autologous dendritic cell vaccine in newly diagnosed glioblastoma. . J Transl Med. 2018 May 29;16(1):142
12. Everson RG, Antonios JP, Liau LM.Cell-Based Immunotherapy of Gliomas.Prog Neurol Surg. 2018;32:90-100
13. Cao JX, Zhang XY, Liu JL, Li D, Li JL, et al. Clinical efficacy of tumor antigen pulsed DC treatment for high-grade glioma patients: evidence from a metaanalysis. PLoS One. 2014;9:e107173.

14. Eagles ME1, Nassiri F2,3, Badhiwala JH et al.Dendritic cell vaccines for high-grade gliomas.Ther Clin Risk Manag. 2018 Jul 26;14:1299-1313.
15. Wang X, Zhao HY, Zhang FC,et al.Dendritic cell-based vaccine for the treatment of malignant glioma: a systematic review.Cancer Invest. 2014 Nov;32(9):451-7
16. Dunn GP, Fecci PE, Curry WT Cancer immunoediting in malignant glioma. Neurosurgery. 2012 Aug;71(2):201-22
17. Medikonda R, Dunn G, Rahman M, Fecci P, Lim M. A review of glioblastoma immunotherapy.J Neurooncol. 2020 Apr 6.
18. de Rosa F, Ridolfi L, Fiammenghi L,et al. Dendritic cell vaccination for metastatic melanoma: a 14-year monoinstitutional experience. Melanoma Res. 2017 Aug;27(4):351-357.
19. Bulgarelli J, Tazzari M, Granato et al. Dendritic Cell Vaccination in Metastatic Melanoma Turns "Non-T Cell Inflamed" Into "T-Cell Inflamed" Tumors. Front Immunol. 2019 Oct 9;10:2353
20. Siesjö P, Visse E, Sjögren HO. Cure of established, intracerebral rat gliomas induced by therapeutic immunizations with tumor cells and purified APC or adjuvant IFN-gamma treatment. J Immunother Emphasis Tumor Immunol. 1996 Sep;19(5):334-45.
21. Liau LM, Black KL, Prins RM, et al Treatment of intracranial gliomas with bone marrow-derived dendritic cells pulsed with tumor antigens. J Neurosurg. 1999 Jun;90(6):1115-24.
22. Ridolfi L, Petrini M, Granato AM,et al .Low-dose temozolomide before dendritic-cell vaccination reduces (specifically) CD4+CD25++Foxp3+ regulatory T-cells in advanced melanoma patients. J Transl Med. 2013 May 31;11:135
23. Wen PY, Macdonald DR, Reardon DA et al;. Updated response assessment criteria for high-grade gliomas: response assessment in neuro-oncology working group. J Clin Oncol. 2010 Apr 10;28(11):1963-72
24. Benjamin M. Ellingson, Patrick Y. Wen, Timothy F. Cloughesy; “Modified Criteria for Radiographic Response Assessment in Glioblastoma Clinical Trials” Neurotherapeutics (2017) 14:307–320 DOI 10.1007/s13311-016-0507-6.
25. Stefanie C. Thust, Martin J. van den Bent, Marion Smits;” Pseudoprogression of brain tumors” . Journal of Magnetic Resonance Imaging 2018;48:571–589.
26. Benjamin M. Ellingson, Caroline Chung, Whitney B. Pope, Jerrold L. Boxerman, Timothy J. Kaufmann; “Pseudoprogression, radionecrosis, inflammation or true tumor progression? challenges associated with glioblastoma response assessment in an evolving therapeutic landscape” J Neurooncol (2017) 134:495–504 DOI 10.1007/s11060-017-2375-2.
27. Marcel T. H. Oei, Frederick J. A. Meijer, Jan-Jurre Mordang, Ewoud J. Smit, Albert J. S. Idema, Bozena M. Goraj, Hendrik O. A. Laue, Mathias Prokop, Rashindra Manniesing:” Observer variability of reference tissue selection for relative cerebral blood volume measurements in glioma patients” European Radiology (2018) 28:3902–3911 https://doi.org/10.1007/s00330-018-5353-y.

APPENDIX A Performance Status Criteria

ECOG Performance Status Scale		Karnofsky Performance Scale	
**Grade**	**Descriptions**	**Percent**	**Description**
**0**	Normal activity. Fully active, able to carry on all pre-disease performance without restriction.	**100**	Normal, no complaints, no evidence of disease.
		**90**	Able to carry on normal activity; minor signs or symptoms of disease.
**1**	Symptoms, but ambulatory. Restricted in physically strenuous activity, but ambulatory and able to carry out work of a light or sedentary nature (e.g., light housework, office work).	**80**	Normal activity with effort; some signs or symptoms of disease.
		**70**	Cares for self, unable to carry on normal activity or to do active work.
**2**	In bed <50% of the time. Ambulatory and capable of all self-care, but unable to carry out any work activities. Up and about more than 50% of waking hours.	**60**	Requires occasional assistance, but is able to care for most of his/her needs.
		**50**	Requires considerable assistance and frequent medical care.
**3**	In bed >50% of the time. Capable of only limited self-care, confined to bed or chair more than 50% of waking hours.	**40**	Disabled, requires special care and assistance.
		**30**	Severely disabled, hospitalization indicated. Death not imminent.
**4**	100% bedridden. Completely disabled. Cannot carry on any self-care. Totally confined to bed or chair.	**20**	Very sick, hospitalization indicated. Death not imminent.
		**10**	Moribund, fatal processes progressing rapidly.
**5**	Dead.	**0**	Dead.

APPENDIX B NCI Common Terminology Criteria for AE

See https://ctep.cancer.gov/protocolDevelopment/electronic_applications/ctc.htm#ctc_50

APPENDIX C Schedule of procedures

Schedule of Study Procedures and Assessments																	
**Period/ Procedure**	**Pre Screening**	**Screening**	**Induction Phase**				**Maintenance Phase [14]**									**EOT**	**FU**
**Study week**		***-28 to 0 (registr. date)***	**1**	**2**	**3**	**4**	**5**	**7**	**9**	**11**	**13**	**15**	**…**	**…**		**(28+-7 days of last treatment)**	**1 year (every 3 months +-14 days)**
Pre-screening informed consent	x																
main study informed consent		x[1]															
AE assessment			x	x	x	x	x	x	x	x	x	x	x	x		x	x [11]
Concomitant medications		x	x	x	x	x	x	x	x	x	x	x	x	x		x	
Leukapheresis	x[2]																
Standard Radiochemoth. (Stupp)	x																
**Study Treatment Administration**																	
Vaccine			x	x	x	x		x		x		x		x			
TMZ [3]							x		x		x		x				
**Clinical procedures**																	
Physical exam		x	x	x	x	x	x	x	x	x	x	x	x	x		x	x
Vital signs		x	x	x	x	x	x	x	x	x	x	x	x	x		x	x
Medical history		x															
Performance status		x	x	x	x	x	x	x	x	x	x	x	x	x		x	x
DTH		x						x				x				x	
**Laboratory procedures**																	
Hematology [4]	x	x [13]	x	x	x	x	x	x	x	x	x	x	x	x		x	
Blood chemistry [5]		x [13]	x	x	x	x	x	x	x	x	x	x	x	x		x	
Coagulation [6]		x [13]	x	x	x	x	x	x	x	x	x	x	x	x		x	
blood sampling for secondary and translational endpoints	x[15]	x [12]						x				x		x		x	x
Serologic markers [7, 10]	x																
HLA characteriz. (OPTIONAL)		x															
Pregnancy test (HCG) [8]		x					x		x		x		x				
**Imaging procedures**																	
Imaging (brain MRI)[9]								x				x		x		x	x

12-lead ECG echocardiogr.[10]	x															

1. Informed consent should be signed within CI 28 days from registration date.
2. Post-surgery, within 28 days from pre-screening informed consent and before standard radiochemotherapy (Stupp). In case of vaccine shortage, additional leukapheresis should be planned and performed in a timely manner to ensure the respect of the interval between cycles as specified by the protocol (i.e. 28±7 days).
3. From day 1 to 5 q28.
4. Including haemoglobin, haematocrit, platelet count, white blood cell count with absolute neutrophil count and absolute lymphocyte count.
5. Including glucose, creatinine, creatinine clearance, urea, bilirubin, AST, ALT, alkaline phosphatase, γGT, sodium, potassium, calcium, chloride, magnesium, total protein, albumin
6. Including PT/PTT/INR.
7. Serologic markers: serum hepatitis assessment, including Hepatitis B surface antigen (HBsAg), Hepatitis B surface antibody (HBsAb), Hepatitis B core antibody (HBcAb), Hepatitis C virus RNA; HIV and treponema pallidum.
8. Only for women of childbearing potential. A serum pregnancy test is strongly encouraged; however, urine pregnancy test is also acceptable.
9. Disease-specific staging criteria (RANO criteria) associated to Perfusion MRI by DSC (dynamic susceptibility contrast) . Restaging will occur every 2 cycles of study treatment during maintenance phase. If there is a questionable pseudoprogression report, the PI or sub-investigators may decide to continue study treatment pending confirmation of progression with subsequent timepoint MRIs if there are no signs and symptoms indicating clear disease progression. The neuroradiologist will distinguish between real progression of GBM from radionecrosis and pseudo progression by evaluating the 5 ROIs of the perfusion study and correlate them with RANO imaging criteria
10. To be repeated if a new leukapheresis will performed.
11. Limited to AE considered related to study drugs.
12. Plus paraffin embedded tissue sections from the primary/metastatic tumor sample.
13. These examinations should be performed within 10 days from registration date.
14. The combined maintenance treatment will continue until disease progression, unacceptable toxicity or withdrawal of consent by the patient, or up to a maximum of 1 year of treatment
15. N° 5 sample tube (10 ml of blood) containing EDTA for PBMCs isolation and N° 1 sample tube (4 ml of blood) containing EDTA for plasma isolation.

APPENDIX D Response Criteria

The Response Assessment in Neuro-Oncology (RANO) guidelines are available here: https://pubmed.ncbi.nlm.nih.gov/26065612/

APPENDIX E World Medical Association Declaration of Helsinki

The current Declaration of Helsinki can be found on the World Medical Association web page via the link provided below:

https://www.wma.net/policies-post/wma-declaration-of-helsinki-ethical-principles-for-medical-research-involving-human-subjects/
